# LIPH contributes to glycolytic phenotype in pancreatic ductal adenocarcinoma by activating LPA/LPAR axis and maintaining ALDOA stability

**DOI:** 10.1186/s12967-023-04702-6

**Published:** 2023-11-21

**Authors:** Lijie Han, Yongsheng Jiang, Minmin Shi, Lina Gan, Zhichong Wu, Meilin Xue, Youwei Zhu, Cheng Xiong, Ting Wang, Xiaozhu Lin, Baiyong Shen, Lingxi Jiang, Hao Chen

**Affiliations:** 1 Department of General Surgery, Pancreatic Disease Center, Ruijin Hospital, Shanghai Jiaotong University School of Medicine, 197 Ruijin 2Nd Road, Shanghai, 200025 China; 2 Research Institute of Pancreatic Diseases, Shanghai Jiaotong University School of Medicine, Shanghai, China; 3 State Key Laboratory of Oncogenes and Related Genes, Shanghai, China; 4 Institute of Translational Medicine, Shanghai Jiaotong University, Shanghai, China; 5 Department of Pathology, Ruijin Hospital, Shanghai Jiao Tong University School of Medicine, Shanghai, China; 6 Department of Nuclear Medicine, Ruijin Hospital, Shanghai Jiaotong University School of Medicine, 197 Ruijin 2Nd Road, Shanghai, China

**Keywords:** LIPH, LPA/LPAR, ALDOA, Glycolysis, Pancreatic cancer

## Abstract

**Background:**

LIPH, a membrane-associated phosphatidic acid-selective phospholipase A1a, can produce LPA (Lysophosphatidic acid) from PA (Phosphatidic acid) on the outer leaflet of the plasma membrane. It is well known that LIPH dysfunction contributes to lipid metabolism disorder. Previous study shows that LIPH was found to be a potential gene related to poor prognosis with pancreatic ductal adenocarcinoma (PDAC). However, the biological functions of LIPH in PDAC remain unclear.

**Methods:**

Cell viability assays were used to evaluate whether LIPH affected cell proliferation. RNA sequencing and immunoprecipitation showed that LIPH participates in tumor glycolysis by stimulating LPA/LPAR axis and maintaining aldolase A (ALDOA) stability in the cytosol. Subcutaneous, orthotopic xenograft models and patient-derived xenograft PDAC model were used to evaluate a newly developed Gemcitabine-based therapy.

**Results:**

LIPH was significantly upregulated in PDAC and was related to later pathological stage and poor prognosis. LIPH downregulation in PDAC cells inhibited colony formation and proliferation. Mechanistically, LIPH triggered PI3K/AKT/HIF1A signaling via LPA/LPAR axis. LIPH also promoted glycolysis and de novo synthesis of glycerolipids by maintaining ALDOA stability in the cytosol. Xenograft models show that PDAC with high LIPH expression levels was sensitive to gemcitabine/ki16425/aldometanib therapy without causing discernible side effects.

**Conclusion:**

LIPH directly bridges PDAC cells and tumor microenvironment to facilitate aberrant aerobic glycolysis via activating LPA/LPAR axis and maintaining ALDOA stability, which provides an actionable gemcitabine-based combination therapy with limited side effects.

**Graphical Abstract:**

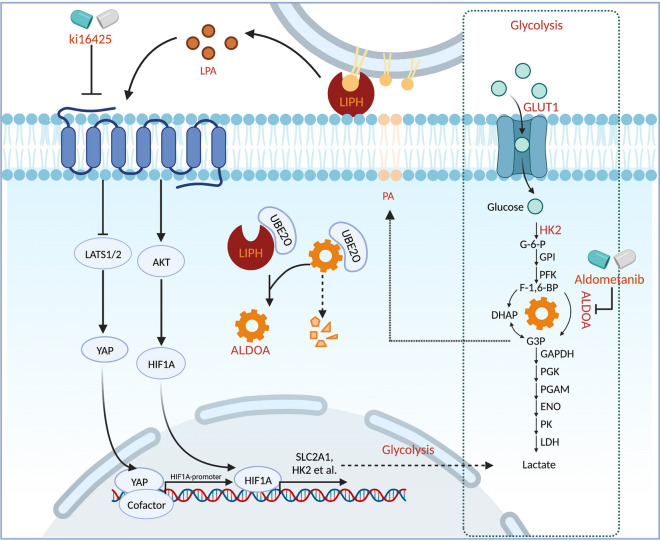

**Supplementary Information:**

The online version contains supplementary material available at 10.1186/s12967-023-04702-6.

## Introduction

Genetic mutation [1], aberrant coding and non-coding RNA expression [2–4], and tumor metabolism [5] contribute to cancer initiation and progression. Pancreatic Ductal Adenocarcinoma (PDAC) is one of the most common human malignancies with a poor prognosis and is the fourth leading cause of cancer-related deaths in the world, with a 5-year survival rate of 10% [6]. PDAC cells prefer aerobic glycolysis to sustain rapid ATP production, rather than the more energetically efficient oxidative phosphorylation, even in the presence of oxygen [7]. Despite prominent glycolysis, the metabolic plasticity of cancer cells, whereby several intermediate enzymes and products from glycolysis participate in multiple alternative pathways, or scavenge nutrients from the environment, constrains the efficiency of monotherapy targeting glycolysis [8].

Distinctively profound matrix deposition in the PDAC microenvironment physically constrains vascularization and impedes the acquisition of serum-derived nutrients [9]. To alleviate this metabolic crisis, PDAC cells can maintain their proliferation by stimulating paracrine and autocrine metabolite transfer as well as by scavenging extracellular lipids via enzymes such as cell-surface proteins (SPs) and exocrine proteins [10]. SPs directly bridge PDAC with the tumor microenvironment (TME), facilitating proliferation signals [11], immune evasion [12] and metabolism reprogramming [13]. LIPH (Lipase H), a membrane-associated phosphatidic acid-selective phospholipase A1a, can produce LPA from PA on the outer leaflet of the plasma membrane, activate the LPA receptor (LPAR), and promote hair follicle development in a paracrine and autocrine manner [14]. Previous studies have shown that LPA is involved in YAP/TAZ-induced gene expression, angiogenesis, cell proliferation and migration [15–18]. However, how LIPH reprograms PDAC metabolic plasticity to sustain rapid proliferation remains elusive.

Aldolase, the fourth enzyme in the glycolysis process, converts fructose 1,6-bisphosphate (FBP) into glycerol-3-phosphate and dihydroxyacetone phosphate, which can fuel glycolytic flow, promote de novo synthesis of glycerolipids, and maintain the LPA/PA reservoir in the intracellular counterpart and cell membrane [19]. The small-molecule aldometanib, an aldolase inhibitor, selectively activates the lysosomal pool of AMPK by blocking FBP binding to aldolase, simulating a cellular state of glucose starvation. Notably, in a preclinical trial, aldometanib alleviated fatty liver disease, reduced fat mass, and extended the lifespan of mice without causing discernible side effects [20]. However, its potential therapeutic effect on malignant tumors, especially PDAC, requires further investigation.

In this study**,** by screening for potential SPs, we found that LIPH may contribute to metabolic plasticity by enhancing glycolysis through LPA/LPAR-mediated nuclear localization of YAP1 and HIF1A. The cytosolic distribution of LIPH maintained ALDOA stability through competitive binding with UBE2O. An LPAR inhibitor combined with aldometanib and gemcitabine efficiently constrained tumor growth in orthotopic xenograft and patient tumor-derived xenograft (PDX) models in mice without significant side effects. Collectively, our results indicate that LIPH may be a promising therapeutic target in patients with PDAC.

## Methods and materials

### Cell culture and regents

Human PDAC cell lines AsPC-1, BxPC-3, CFPAC-1, PANC-1, PNAC-1-luc, MIA PaCa-2, 8988t, 8988s, mouse PDAC cell lines Panc02, and KPC1199, and human normal pancreatic duct cells (HPNE) were all preserved at the Pancreatic Disease Center, Ruijin Hospital, School of Medicine, Shanghai Jiao Tong University. Doxycycline-inducible KRAS^G12D^ BxPC-3 cells were gifted by Pro. Sun (Shanghai Cancer Institute, Renji Hospital, School of Medicine, Shanghai Jiao Tong University). All cells were grown in the suggested culture medium, supplemented with 10% fetal bovine serum (FBS, Gibco), 100 U/mL penicillin and 100 μg/mL streptomycin at 37 ℃ with 5% CO_2_. A final concentration of 2.5% horse serum was added to MIA PaCa-2 cultured cells. The base medium for AsPC-1 and BxPC-3 cells is RPMI-1640 Medium (Meilunbio, China); for CFPAC-1, Iscove's Modified Dulbecco’s Medium (IMDM) (BIOAGRIO, USA); for PANC-1, PANC-1-luc, MIA PaCa-2, 8988t, 8988s, Panc02, and KPC1199; and for HPNE, Dulbecco's Modified Eagle's Medium (DMEM) (Meilunbio, China). All cells were checked routinely for mycoplasma contamination. U0126, Ki16425, aldometanib, gemcitabine, MG132 and cycloheximide were purchased from MCE (Shanghai, China). LPA and PA were purchased from Sigma-Aldrich. Doxycycline was purchased from TargetMol (Shanghai, China), and 1 μg/mL doxycycline was added to induce the expression of KRAS^G12D^.

### Transfection

Plasmid transfections were performed using Lipofectamine^®^2000 (Invitrogen, Carlsbad, CA, USA) following the manufacturer’s instructions. Specific luciferase plasmids, shRNAs, vector controls, and full-length cDNA encoding human LIPH were synthesized by BioGene (Shanghai, China). For lentivirus packaging, 293 T cells were transfected using lipofectamine 2000 with 2 μg of each plasmid and 2 μg of helper plasmid (1 μg pMD2G and 1 μg of psPAX2). Viral Supplements were collected 48 h later and filtered with a 0.22 μm filter. PDAC cells, 3 × 10^5^, were infected with viral supernatants and 10 μg/mL polybrene (Yeasen, China) for 6 h. Transduced cells were selected with 10 μg/mL puromycin (Meilunbio; China) to produce stable cell lines. Further information on plasmids is described in the Extended Information (Additional file 8).

### In vitro functional assays for tumorigenicity

For the cell proliferation assay, cells were seeded in 96­well plates at a density of 1 × 10^3^ cells/well. Cell Counting Kit-8 (CCK-8) (Meilunbio, China) was used according to the manufacturer’s instructions, and the optical density at 450 nm was measured (Biotek, USA). To exclude the effect of LPA in fetal bovine serum, one day after seeding, cells were changed to DMEM, and viability was measured at 72 h. To evaluate the effects of PA and LPA, PDAC cells were seeded at 3 × 10^3^ cells/well. One day after seeding, cells were changed to DMEM containing 0.5% FAF-BSA (starvation medium) (Beyotime, China) and incubated for 24 h. After starvation, cells were incubated in 0.5% FAF-BSA, 0.5% FAF-BSA along with 10 µM PA or 0.5% FAF-BSA along with 10 µM LPA. Cell viability was assayed at the indicated time points. For the colony formation assay, cells were seeded in 6­well plates at a density of 1 × 10^3^ cells/well for 10 days, followed by staining of crystal violet (Beyotime, China) dye for 20 min.

### Flow cytometric analysis

For the apoptosis assay, PC cells were seeded in 6-well plates at a density of 1 × 10^5^. After starvation or incubation with gemcitabine (1 μg/mL) for three days, cells were collected, resuspended, and incubated with fluorescein isothiocyanate-conjugated annexin V and propidium iodide (Vazyme, China) for 30 min in the dark at room temperature, and analyzed by flow cytometry (CytoFLEX5, Beckman).

### Subcutaneous xenograft model, orthotopic xenograft model and PDX model

For the subcutaneous xenograft model, PANC-1 (2 × 10^6^ cells per mouse), CFPAC-1 (2 × 10^6^ cells per mouse), or MIA PaCa-2 (2 × 10^6^ cells per mouse) were injected into the right or left dorsal flanks of BALB/c nude mice. The tumor volumes were measured three times per week and calculated according to the formula V(volume) = 0.5 × L(length) × W(width)^2^. After two weeks, the mice were euthanized and the tumors were collected for histological and immunohistochemical (IHC) analysis. To perform 18F-fluorodeoxyglucose (18F-FDG) PET/CT, the mice were anesthetized with sodium pentobarbital (10 mg/kg) and intravenous 18F-FDG was administered. One hour later, PET/CT images were obtained using an Inveon Animal PET/CT scanner combined with three-dimensional ordered-subset expectation maximization (OSEM3D)/maximum algorithms. The SUV_max_ within the tumor was used for further analysis.

An orthotopic xenograft model was constructed by injecting human luciferase-tagged PANC-1 cells (PANC1-Luc; 1 × 10^6^ cells per mouse). After four weeks, the mice were intraperitoneally injected with d-luciferin potassium (150 mg/kg) and analyzed using an IVIS Spectrum Imaging System (Tanon, China). Imaging was performed weekly to closely monitor tumor proliferation and drug efficiency. Tumors and adjacent areas were collected at the end of the experiment.

To construct a patient-derived xenograft (PDX), PDAC tissues from two individuals, which had been checked and confirmed by two pathologists, were used. Briefly, the surgical specimen from pancreatectomy was washed with P/S solutions, carefully cut into 1 mm^3^, stored at − 80 °C with freezing medium (10% DMSO, 90% FBS) and buried in the right or left dorsal flank of NGS mice within one week to generate first generation (F1) tumor. When the tumor reached 800 mm^3^, the mice were sacrificed, and the tumor was cut into 1 mm^3^ for further transplantation. The relevant drugs were administered to the tumors as soon as their volume reached approximately 100–150 mm^3^ (6–8 weeks after transplantation). The control group received physiological saline solution via intraperitoneal injection (i.p.), whereas the other four groups were treated with gemcitabine (50 mg/kg i.p., twice a week), ki16425 (4 mg/kg i.p., qod), or aldometanib (5 mg/kg, i.g., qod). The mice were sacrificed after two weeks of treatment.

### Metabolism analysis

Metabolic assays for the extracellular acidification rate (ECAR) and oxygen consumption rate (OCR) were performed using a Seahorse XF96 Flux Analyzer (Agilent, USA) according to the manufacturer’s instructions. Briefly, 2 × 10^5^ cells were seeded in XF 96-well plates. One day after seeding, cells were changed to DMEM containing 0.5% FAF-BSA and incubated for 24 h. After starvation, cells were incubated in 0.5% FAF-BSA, 0.5% FAF-BSA along with 10 µM PA or 0.5% FAF-BSA along with 10 µM LPA for 6 h. One hour before testing, the medium was replaced with the assay medium. For the ECAR test, 10 mmol/L glucose, 1 mmol/L oligomycin, and 50 mmol/L 2-deoxyglucose (2-DG) were injected into each well. For OCR test, 1 μmol/L oligomycin, 2 μmol/L FCCP, 0.5 μmol/L rotenone, and 0.5 mmol/L actinomycin A were added to the wells. For ATP production assays, cells were grown overnight in 6-well plate culture dishes, followed by treatment with the indicated reagents. The cells were lysed and clarified by centrifugation, and the supernatants were extracted for BCA and ATP measurements (Beyotime, China).

### Histology and immunohistochemistry (IHC)

Hematoxylin and eosin (H&E) staining was performed routinely [21] and observed with GRUNDIUM OCUS^®^ (Grundium, Finland). For IHC, paraffin sections were dewaxed with an ethanol gradient, steam-heated for antigen retrieval in citrate-based buffer for 20 min, blocked with 3% BSA diluted in Tris-buffered saline plus 0.1% Tween 20, and stained with primary and appropriate secondary antibodies to detect the corresponding proteins. The tissue staining was scored negative when < 5% tumor cells showed expression. Positive scores (+–+++) were based on the percentage of tumor cells and staining intensity within the tumor sample. Information on the antibodies is provided in the Extended Information section.

### Immunofluorescence (IF)

To assess the distribution of LIPH, ALDOA, and YAP1, cells (CFPAC-1, PANC-1, and MIA PaCa-2) were seeded on no. 1.5 cover glass (Corning, USA). Cells were fixed with 4% paraformaldehyde (Servicebio, China) for 10 min, washed, and permeabilized with 0.25% Triton-X100 (Sigma, USA) for 5 min. Cells were washed with ice-cold PBS, blocked and incubated for 30 min in blocking solution (PBS, 1% BSA, 0.1% Tween 20, 22.52 mg/mL glycine), followed by incubation with primary antibodies in dilution solution (PBS, 1% BSA, 0.1% Tween 20) for 1 h at 37 °C. After three washes of 5 min in PBS, the cells were incubated with goat anti-rabbit antibody or goat anti-mouse antibody for 30 min at room temperature. Cells were washed three times for 5 min in PBS, with the first wash containing 0.1 μg/mL DAPI (Thermo Scientific; USA). IF staining was performed on PDAC tissues from archived formalin-fixed paraffin-embedded tumors according to standard protocols [22]. Samples were observed under a confocal laser scanning microscope (Zeiss LSM900, Germany). Information on the antibodies is provided in the “Extended information”.

### Immunoblotting and antibody

Cell lysates were prepared using an ice-cold RIPA lysis buffer (Solarbio, China) supplemented with a phosphatase inhibitor cocktail (NCM; China). After total protein was normalized by BCA assay (Invitrogen, USA), protein samples were separated by 8–10% SDS-PAGE gel electrophoresis and transferred onto PVDF membranes. After blocking with 5% BSA (Proliant, New Zealand) diluted in Tris-buffered saline plus 0.1% Tween 20 for 1 h at room temperature, membranes were incubated overnight with primary antibody and 1 h with secondary antibody (Proteintech, 1:10,000). Enhanced chemiluminescence (ECL) was performed using an ECL kit (Meilunbio, China) and visualized using the Tanon system (Shanghai, China). Information on the antibodies is provided in the Additional files. Nuclear and Cytoplasmic Protein Extraction Kit (Beyotime, China) was used to extract the nuclear and cytoplasmic proteins.

For immunoprecipitation, protein lysates were prepared in ice-cold IGEPAL^®^ CA-630 buffer (Beyotime; China) supplemented with an inhibitor cocktail (NCM, China). The cells were mechanically ruptured at least five times using a cell scraper (Corning, USA). Lysates were then centrifuged at 12000 rpm/min, 4 °C for 15 min, and the supernatant was collected. The samples were incubated with protein A/G beads (Beyotime; China) for 1 h at 4 °C to reduce nonspecific binding. The samples were centrifuged, and the supernatant was collected for immunoprecipitation. The supernatant was incubated with FLAG antibody (14793, Cell Signaling Technology), ALDOA antibody (11217-1-AP, Proteintech), and protein A/G beads overnight at 4 °C with gentle rocking. After that, the samples were centrifuged at 4 °C and the beads were washed with lysis buffer three times. The beads were reconstituted in Non-Reducing Lane Marker Sample Buffer (39001, Thermo Scientific) and boiled for 5 min before immunoblotting.

### RNA extraction and quantitative reverse transcriptase PCR

Total RNA was extracted using an RNA Extraction Kit (Accurate Biology, China), and reverse transcription was performed using an Evo M-MLV RT-PCR Kit (Accurate Biology, China) according to the manufacturer’s instructions. For quantitative PCR analysis, double-stranded cDNA was amplified using a SYBR Green PCR Kit (Accurate Biology, China) and detected using qTOWER384G (Analytik Jena, Germany). The primers used in this study are listed in the Extended Information.

### RNA-seq and read mapping

For RNA-seq, total RNAs were isolated using an RNA Extraction Kit (Accurate Biology, China) following the manufacturer’s instructions and sent to Xurangene Co., Ltd. Clustering and sequencing were then performed. The mapping results were processed using custom scripts, and all samples were prepared in triplicate. The detailed methodology for transcript assembly and statistical analysis has been described in a previous study [23].

### LC–MS

Gel fragments were cut from SDS PAGE. The in-gel proteins were reduced, alkylated, and digested with 12.5 ng/μL trypsin in 25 mM NH_4_HCO_3_ overnight. The peptides were then collected with solution (60% ACN /0.1% TFA), pooled, and dried entirely in a vacuum centrifuge.

LC–MS analysis was carried out on a Q-Exactive mass spectrometer (Thermo Fisher Scientific, USA) in positive ion mode, coupled to an Easy nLC (Thermo Fisher Scientific, USA) for 60 min. Survey scans (300–1800 *m*/*z*) were taken at a resolution of 70,000 at *m*/*z* 100, the AGC target was 1e6, the maximum inject time was 50 ms, the number of scan ranges was 1, and the dynamic exclusion duration was 30 s. The peptide recognition mode was used to operate the instrument. MS data were obtained using the data-dependent Top20 method. The resolution of the HCD spectra was 17,500 at *m*/*z* 100, AGC target was 1e5, isolation width was 1.5 *m*/*z*, microscans were 1, and the maximum injection time was 50 ms.

For data analysis, MS/MS spectra were searched using Proteome Discoverer 1.4 against uniprot_Homo_sapiens_203800_20220104. Protein identification options: Enzyme = trypsin; maximum missed cleavage = 2; fixed modification = carbamidomethyl (C); dynamic modification = oxidation (M); filter by peptide confidence = high.

### Chromatin immunoprecipitation (ChIP)

ChIP experiments were conducted according to the standard protocols [21]. In brief, MIA PaCa-2 cells stably transfected with NC-vector or OE-LIPH plasmid were cross-linked with 1% formaldehyde for 10 min at room temperature and then quenched with glycine (Thermo Fisher Scientific, USA). Appropriate DNA fragments, which were then incubated with YAP1 antibodies, were obtained by carefully sonicating the nuclear lysates. Normal rabbit IgG was used as the negative control. The primers used for ChIP are listed in the Extended Information.

### Construction and visualization of docking mode

To conduct docking [24], proteins with full-length sequences were collected from UniProt (https://www.uniprot.org/). Input Receptor Molecule: ALDAO (P04075). Input ligands: LIPH (Q8WWY8) or UBE2O (Q9C0C9).

Protein–protein docking was based on a hybrid algorithm of template-based modeling and ab initio-free docking (using a hybrid docking strategy). The metrics of ALDAO + UBE2O complex (Docking Score: − 206.85; Confidence Score:0.7571; Ligand rmsd (Å):78.10). The metrics of ALDAO + LIPH complex (Docking Score: − 255.10; Confidence Score:0.8911; Ligand rmsd (Å):61.20).

Superposition of the structures was performed using CCP4. Visualizations of the atomic and cartoon/surface models were performed using PyMOL (PyMOL Molecular Graphics System, version 2.0; Schrödinger, LLC).

### Bioinformatics analysis

The Gene Expression Omnibus (GEO) datasets GSE15471, GSE16515, GSE62452, and GSE71729 were used to screen potential SPs. Microarray data were normalized and Log2-transformed, and Linear Models for Microarray Data (Limma, v3.17) were used for differential analyses. For TCGA and GTEx data, gene expression data were obtained from the data hubs at UCSC Xena (https://xenabrowser.net/hub/). The scRNA-seq data were downloaded from NGDC (CRA001160) and reanalyzed using Seurat2.0 (http://satijalab.org/seurat/) and Monocle2 (http://cole-trapnell-lab.github.io/monocle-release). Gene set enrichment analysis (GSEA, v4.3.2) was performed on the Broad Institute Platform and Gene set variation analysis (GSVA, v3.17) analysis was performed by “GSVA” R package. The association of LIPH mRNA with clinical prognosis was determined using GEPIA [25] and Kaplan–Meier (http://www.kmplot.com/). Survival Statistical significance (P value) was set at 0.05.

### Study approval

Studies using human tissues were reviewed and approved by the Committee for Ethical Review of Research Involving Human Subjects at the Ruijin Hospital. All patients provided written informed consent for the use of their clinical specimens for medical research. Animal experiments were approved by the review board on the use of living animals at Ruijin Hospital (Shanghai, China). The mice were housed in a pathogen-free laboratory animal unit at PNK (Shanghai, China).

### Statistical analysis

Data are presented as mean ± standard deviation. Student’s t-test was used to compare differences between two groups. One-way ANOVA was performed when multiple conditions were compared for each variable. Two-way ANOVA was used when multiple groups were compared for more than one variable. Survival was analyzed using the log-rank test. All statistical analyses were performed using GraphPad Prism 8.0, Excel and SPSS v23. Differences were considered statistically significant at P < 0.05.

## Results

### Identification of LIPH as a potential oncogene in PDAC

**Fig. 1:**
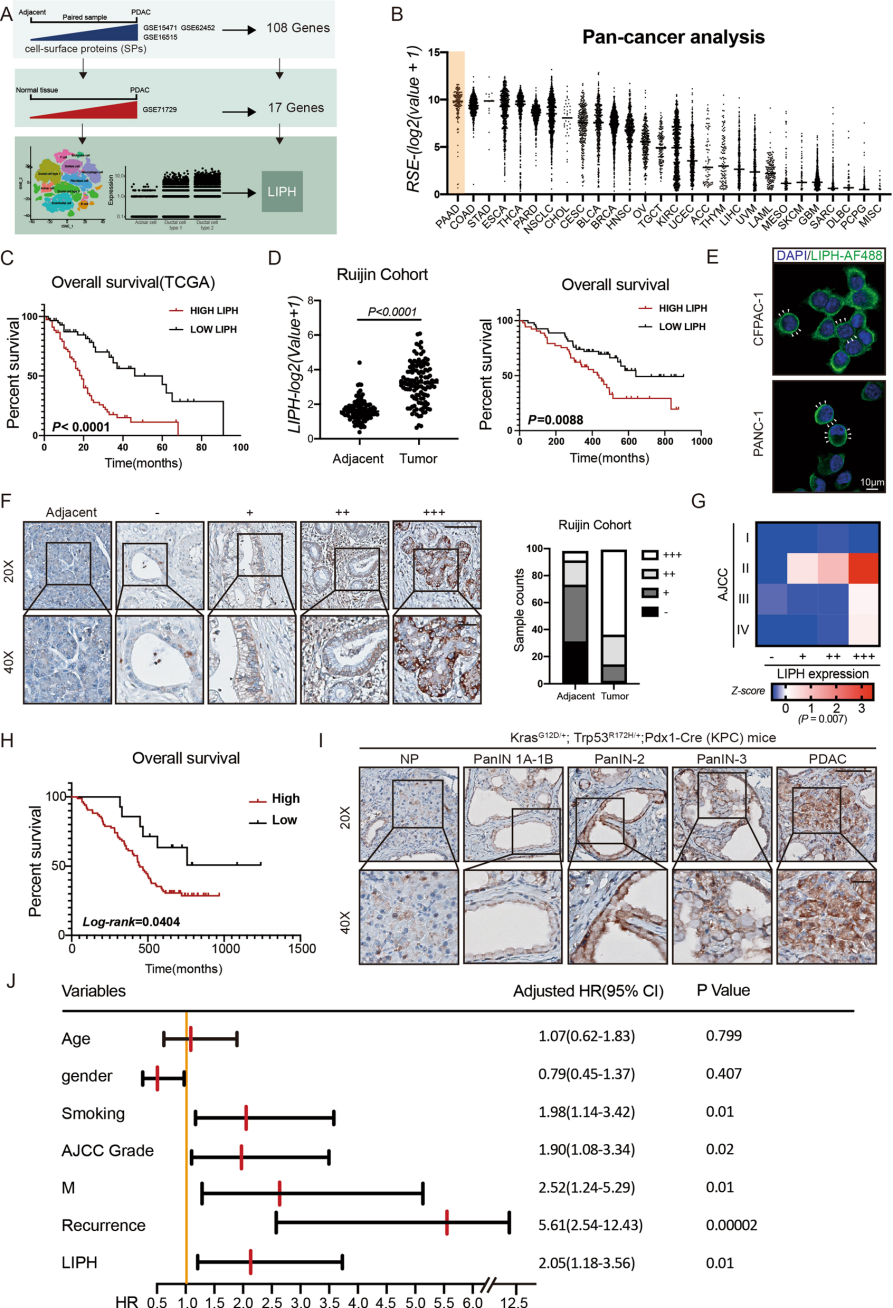
LIPH was significantly upregulated in PDAC and was related to later pathological stage and poor prognosis.** A** Identification of LIPH as a potential oncogene in pancreatic cancer. **B** LIPH expression in PDAC was the highest among 28 cancer types. **C** Kaplan–Meier analysis of overall survival rate related to the expression of LIPH in 177 cases based on the TCGA database (Cutoff: Best Cutoff, High = 59, Low = 118). **D** Kaplan–Meier analysis of overall survival rate related to the expression of LIPH in 107 cases based on the RNA sequencing results of Ruijin patients (Cutoff: median, High = 53, Low = 54). **E** IF staining of LIPH in PANC-1 and CFPAC-1. **F** Standard IHC staining and respective sample count of LIPH expression in 99 pancreatic ductal adenocarcinoma and paired adjacent normal tissues. **G** The heatmap showed that staining count highly correlated with AJCC stage based on TMA. **H** Kaplan–Meier analysis of overall survival rate based on the IHC count (Cutoff: High (+++/++) = 85, Low (±) = 14). **I** Standard IHC staining in different stages of PDAC progression in KPC mice. **J** Univariate Cox regression analysis based on the Ruijin cohort. Scale bar = 100 µm. *P < 0.05, **P < 0.01, ***P < 0.001 and ****P < 0.0001

To identify the potential oncogenes among SPs that play a pivotal role in supporting PDAC with limited vascularization (Fig. 1A), we first analyzed the expression of SPs that were expressed at significantly higher levels in PDAC tissues than adjacent areas in three GEO datasets [26]: GSE15471, GSE16515, and GSE62452 (Cross1, 108 genes) (Additional file 1: Fig. S1A). Among 108 genes, only 17 SPs were upregulated in PDAC compared to multiple non-tumor tissues in GSE71729 (Additional file 1: Fig. S1B). We then investigated the clinical prognosis of these SPs using the PAAD-TCGA database and found that 10 genes were positively correlated with poor prognosis of PDAC (Additional file 7: Table S1). LIPH was mainly expressed in ductal cells and was most prominently upregulated during the transition from abnormal to malignant according to the scRNA-seq data (Additional file 1: Fig. S1C, D, E; Additional file 7: Table S1). Pan-cancer analysis using the expression of 28 tumors and the Cancer Cell Line Encyclopedia (CCLE) revealed that LIPH was highly expressed in PDAC tissues and cell lines originating from pancreatic cancer compared to other tumor types (Fig. 1B; Additional file 1: Fig. S1F). LIPH expression was significantly higher in PDAC tissues than in the adjacent normal tissues in the Ruijin cohort (Fig. 1D), PAAD-TCGA (Additional file 1: Fig. S1G), GEO dataset (GSE15471, GSE16515 and GSE62452, Additional file 1: Fig. S1H). Kaplan–Meier analysis revealed that high LIPH expression in cancer tissues was associated with poor prognosis in patients with PDAC (Fig. 1C, D; Additional file 1: Fig. S1I). Immunofluorescence (IF) staining confirmed the presence of LIPH in the plasma membrane and cytoplasm of PANC-1 and CFPAC-1 cells (Fig. 1E). We validated our results at the protein level by performing immunohistochemistry (IHC) staining in Ruijin tissue microarray (TMA) containing 99 pathologist-verified PDAC specimens with paired corresponding adjacent pancreatic tissues. The staining score was highly correlated with advanced tumor stage (Fig. 1F, G) and survival (Fig. 1H). We also examined LIPH expression patterns in Kras^G12D(^KI/+)/Trp-53^R172H^(KI/+)/Pdx1-Cre (TG/+) (KPC) mice (Fig. 1I). LIPH staining positively correlated with PDAC progression. In addition, univariate Cox regression analysis showed that LIPH expression, smoking, metastasis, American Joint Committee on Cancer (AJCC) stage, and recurrence were significantly associated with overall survival (Fig. 1J). Multivariate Cox regression analysis identified LIPH expression and tumor recurrence as independent predictors of overall survival in patients with PDAC (Additional file 1: Fig. S1J). Taken together, these data indicate that LIPH is an unfavorable prognostic factor for pancreatic cancer and is highly correlated with disease progression.

### LIPH promoted proliferation and inhibited apoptosis of pancreatic cancer cells in vitro

**Fig. 2:**
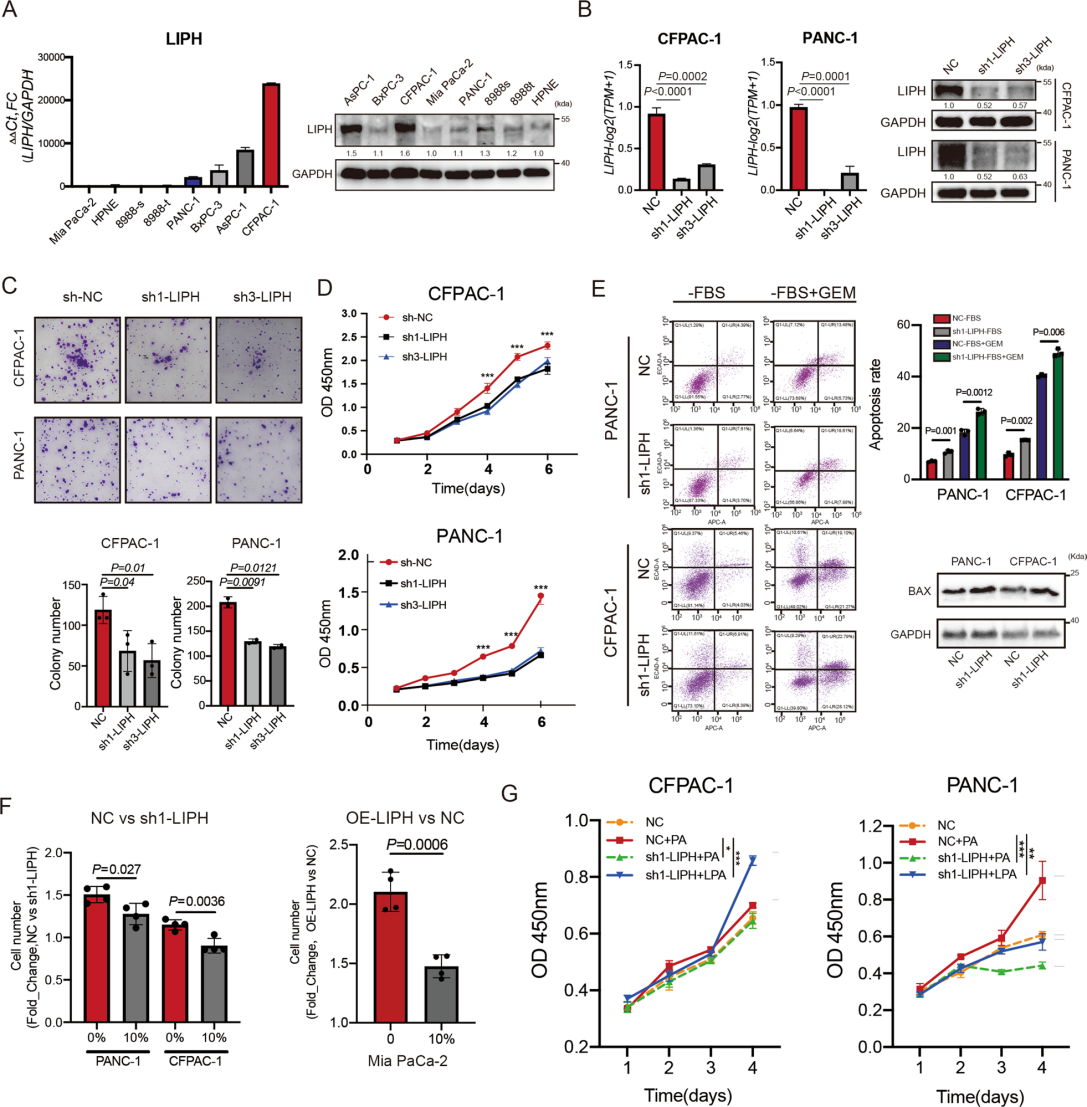
LIPH promoted proliferation and inhibited apoptosis of pancreatic cancer cells in vitro*. A* LIPH expression in pancreatic cancer cell lines (MIA PaCa-2, HPNE, 8988s, 8988t, PANC-1, BxPC-3, AsPC-1 and CFPAC-1) compared with that in normal pancreatic ductal epithelial cell line HPNE, detected by qPCR and Western Blotting. **B** The mRNA and protein expression of LIPH in LIPH-knockdown PANC-1 and CFPAC-1 cells. **C** The colony formation ability was inhibited by LIPH knockdown. **D** The CCK8 assay showed that LIPH knockdown in PANC-1 and CFPAC-1 resulted in suppression of proliferation ability. **E** Flow cytometric apoptosis assay and BAX protein expression (starvation) were used to detect the apoptosis level following LIPH knockdown (−FBS: Medium without FBS; + GEM: Medium with Gemcitabine). **F** After 72 h serum deprivation, the CCK8 assays were used to detect cell proliferation ability. For positive control, 10% FBS was used to culture cancer cells. **G** CFPAC-1 and PANC-1 cells were cultured one day in 0.5% FAF-BSA complete medium (starvation), and then exogenous PA (10 µM) or LPA (10 µM) were added to the cultured cells, followed by CCK8 assays at 72 h to detect cell proliferation ability. *P < 0.05, **P < 0.01, ***P < 0.001 and ****P < 0.0001

To study the effect of LIPH on PDAC, we examined the expression level of LIPH in eight pancreatic cancer cell lines at the mRNA and protein levels, with the highest expression in CFPAC-1 cells and the lowest expression observed in MIA PaCa-2 cells (Fig. 2A). PANC-1 and CFPAC-1 cells were stably transfected with control short hairpin RNA (shRNA) or shRNA (sh1-LIPH and sh3-LIPH) targeting LIPH, and mRNA and protein levels were compared (Fig. 2B). Additionally, the full-length human LIPH cDNA was cloned into lentiviral vectors (OE-LIPH) and transfected into MIA PaCa-2 cells (Additional file 2: Fig. S2B). Gene set variation analysis (GSVA) of the Ruijin cohort showed a significant positive correlation between high LIPH expression and glycolysis, hypoxia, and Hippo signaling pathways (Additional file 2: Fig. S2A). Therefore, we speculate that LIPH is mainly involved in the regulation of pancreatic cancer (PC) cell proliferation and self-renewal. We used the CCK8 assay, colony formation assay, and flow cytometry to measure the effect of LIPH on PC cell proliferation and growth. LIPH knockdown inhibited colony formation and proliferation of PANC-1 and CFPAC-1 cells (Fig. 2C, D). Conversely, LIPH overexpression enhanced colony formation and proliferation of MIA PaCa-2 cells (Additional file 2: Fig. S2C). Knockdown of LIPH expression effectively promoted apoptosis of cancer cells as well as upregulation of the apoptosis-associated protein BAX (Fig. 2E), increasing chemosensitivity to high gemcitabine concentrations (Additional file 2: Fig. S2E), whereas overexpression of LIPH reversed this effect (Additional file 2: Fig. S2D). Serum deprivation during apoptosis suggests a potential role for LIPH in maintaining cancer cell survival by catalyzing PA in a PDAC microenvironment with extremely poor vascularization. Knockdown of LIPH significantly constrained PANC-1 and CFPAC-1 proliferation ability in the serum-deprived medium compared with 10% fetal bovine serum (FBS)-complete medium. Conversely, LIPH overexpression in MIA PaCa-2 cells promoted their proliferation in a serum-deprived medium (Fig. 2F). To evaluate the exact role of LIPH in PDAC without the interference of LPA in FBS, fatty acid-free (FAF)-BSA with an additional supplement was used to maintain cell cultures. The results showed that LIPH knockdown constrained the utilization of exogenous PA, thus alleviating cell proliferation. The addition of LPA to the medium efficiently reversed the effects of LIPH knockdown (Fig. 2G; Additional file 2: Fig. S2F, G).

### LIPH knockdown inhibited xenograft tumor growth

**Fig. 3:**
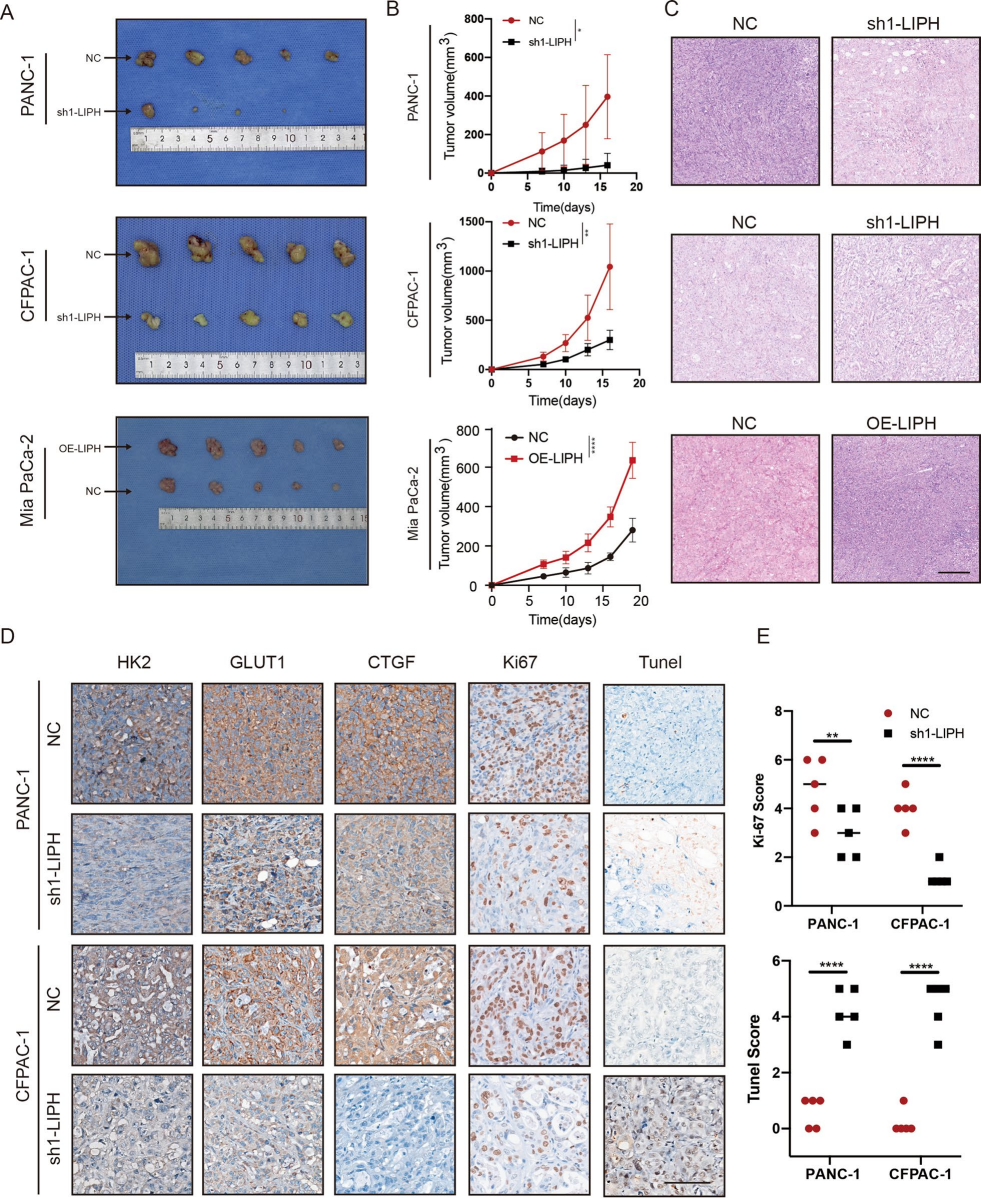
LIPH Knockdown inhibited xenograft tumor growth.** A** Subcutaneous xenograft tumors in BALB/c mice. **B** The tumor was measured every three days after seven days injection and tumors were harvested at a certain size. **C** H&E-stained xenograft tumor tissue. **D** IHC staining of HK2, GLUT1, CTGF, Ki67 and TUNEL. Scale bar = 100 µm. **E** The intensity of ki67 and TUNEL in **C**. *P < 0.05, **P < 0.01, ***P < 0.001 and ****P < 0.0001

To test the tumorigenic ability of LIPH in vivo, PANC-1 and CFPAC-1 cells stably transfected with vector plasmid or sh1-LIPH, or MIA PaCa-2 cells stably transfected with vector and overexpression plasmids were inoculated into the right or left dorsal flank of BALB/c nude mice. Tumor size was carefully measured every three days after seven days injection. IHC was used to further validate transfection efficiency (Additional file 3: Fig. S3A). Tumors formed in the presence of sh1-LIPH were significantly smaller than those formed by the vector control (Fig. 3A). In contrast, LIPH overexpression enhanced tumorigenicity in vivo (Fig. 3A). To validate the ability of LIPH to induce cell proliferation and apoptosis, xenograft tumors were stained with H&E, ki67 and TUNEL. The expression of ki67 was decreased in sh1-LIPH pancreatic cancer tumors, and the apoptosis marker, TUNEL, was significantly increased (Fig. 3D, E). In addition to the downstream genes of the Hippo pathway, CTGF and glycolysis hub genes GLUT1 and HK2 were considerably downregulated in sh1-LIPH xenograft tumors, whereas LIPH overexpression reinstated their expression (Fig. 3D; Additional file 3: Fig. S3C). These results were highly consistent with our in vitro findings that LIPH knockdown inhibited the proliferation and promoted the apoptosis of pancreatic cancer cells in vitro.

### LPA produced by LIPH promoted PDAC progression through enhancing glycolysis

**Fig. 4:**
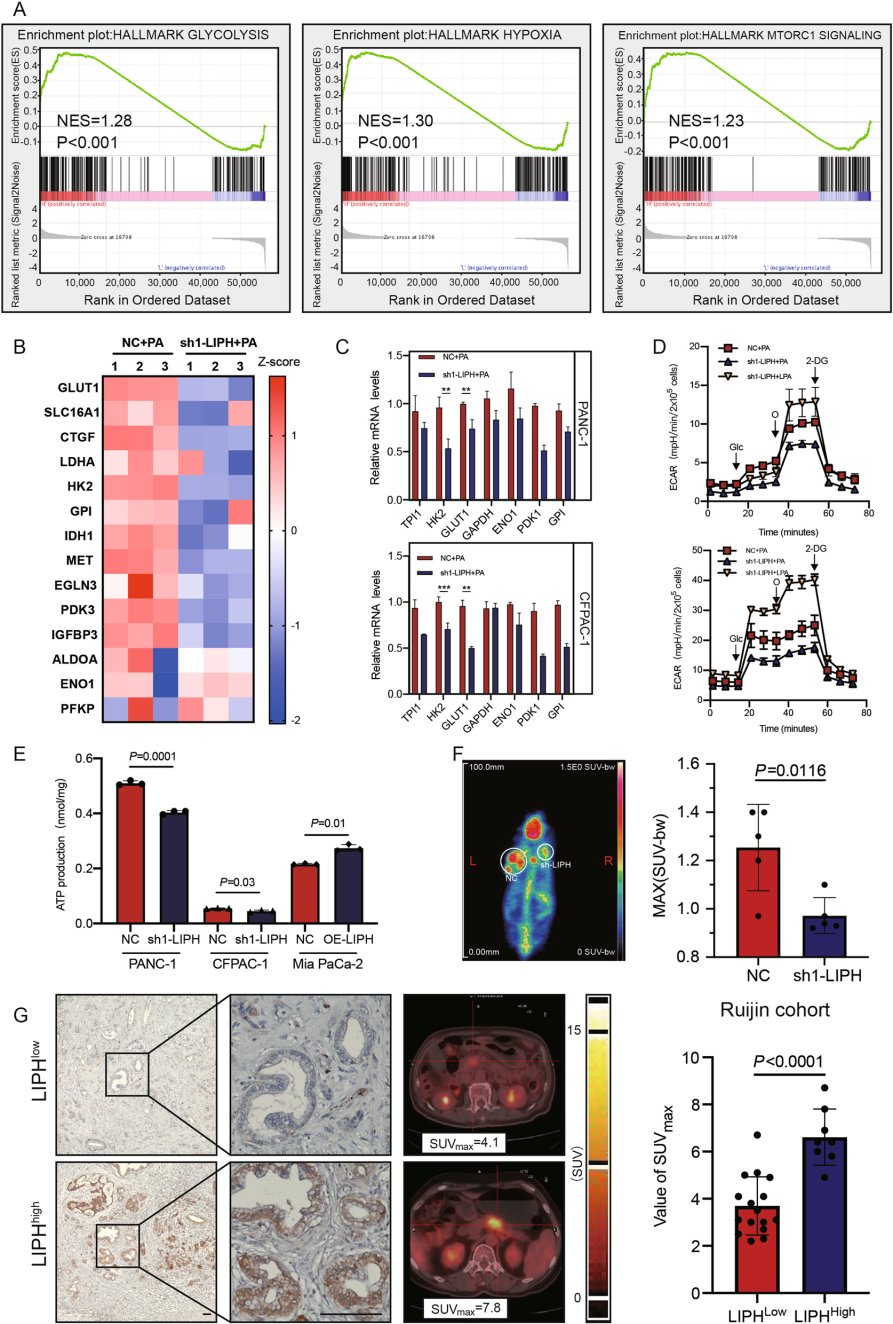
LPA produced by LIPH promoted PDAC progression through enhancing glycolysis.** A** GSEA using hallmark gene sets was performed to compare PANC-1 control and PANC-1 sh1-LIPH-transfected cells treated with PA (10 µM). NES, normalized enrichment score. **B** A heatmap showing the expression of the glycolysis-related genes and hypoxia-related genes in PANC-1 NC and LIPH-knockdown cells in the presence of PA (10 µM). **C** Relative mRNA levels of glycolysis-related genes of PDAC with or without LIPH inhibition in the presence of PA (10 µM) treatment. Expression was detected by q-PCR. **D** ECAR detected by Seahorse analyzer in control and sh1-LIPH-transfected cells (PANC-1 up, CFPAC-1 low). Cells were seeded and cultured one day in 0.5% FAF-BSA complete medium (starvation), and then exogenous PA (10 µM) or LPA (10 µM) was added to culture cell before analysis. **E** ATP production of PC cells with or without LIPH inhibition (Glc: Glucose; O: oligomycin; 2-DG: 2-deoxyglucose). **F** Graph of 18F-FDG uptake in the subcutaneous xenograft model. Control and sh1-LIPH-transfected CFPAC-1 cells were used to establish the subcutaneous xenograft. **G** Representative images of LIPH expression in tumor tissues from PDAC patients who received preoperative 18F-FDG PET/CT examination. The difference in SUV_max_ value between LIPH^high^ patients and LIPH^low^ patients was further analyzed. Scale bar = 100 µm. *P < 0.05, **P < 0.01, ***P < 0.001 and ****P < 0.0001

To gain a comprehensive insight into the mechanism by which LIPH promoted pancreatic cancer cell growth, gene set enrichment analysis (GSEA) was performed in the PAAD-TCGA, GSE15471 and GSE28735. A highly positive correlation between LIPH expression and glycolysis was observed in high-LIPH PDAC tissue samples (Additional file 4: Fig. S4A). The global gene expression of sh1-LIPH PDAC cells compared with control cells after PA treatment was profiled and analyzed using GSEA and KEGG pathway analysis. The results indicated that the differentially expressed genes were mainly related to metabolic processes, including genes associated with glycolysis, hypoxia and MTORC1 signaling, suggesting that LIPH knockdown might restrain metabolic processes in PDAC (Fig. 4A). Expression of several critical genes in the glycolysis and hypoxia pathways, including GLUT1, HK2, IGFBP3, PDK3, EGLN3, and CTGF, was significantly downregulated in sh1-LIPH cells compared to the control cells in the presence of PA (Fig. 4B). Alterations in GLUT1 and HK2 expression were further confirmed using real-time qPCR or IHC (Figs. 4C, 3D). To further investigate glycolysis alternation with the manipulation of LIPH expression, glycolysis stress tests using extracellular acidification rate (ECAR) were performed to evaluate the glycolytic activities in PDAC cells. Exogenous PA effectively enhanced glycolytic capacity and glycolytic reserve in cells transfected with the control vector; however, it had limited influence on LIPH-knockdown cells, and the effect could be reversed by supplementing with exogenous LPA (Fig. 4D, Additional file 4: Fig. S4B). However, overexpression of LIPH in MIA PaCa-2 cells promoted the utilization of exogenous PA (Additional file 4: Fig. S4C). Measurement of basal and maximal respiration also suggested that LIPH overexpression induced a metabolic shift towards glycolysis (Additional file 4: Fig. S4D). LIPH knockdown led to lower ATP production, whereas LIPH overexpression reversed this effect (Fig. 4E).

18F-fluorodeoxyglucose (18F-FDG) PET/CT has been widely used for the visualization of the metabolic activity of tumor cells owing to their high glucose uptake ability. The maximum standardized uptake value (SUV_max_) has been used as a reliable marker for the prognosis of PDAC [27]. To further examine the glycolysis uptake ability in vivo, 18F-FDG PET/CT was performed. As expected, LIPH knockdown significantly inhibited the glucose uptake ability of subcutaneous tumors (Fig. 4F). A systematic analysis of PET/CT images and corresponding paraffin sections from 24 patients at Ruijin Hospital revealed that patients with high LIPH (++/+++) levels exhibited higher SUV_max_, which indicates higher glucose uptake ability, than patients with low LIPH (−/ +) levels (Fig. 4G). Taken together, these data indicated that LPA produced by LIPH promoted pancreatic cancer cell proliferation by enhancing glycolysis in vitro and in vivo.

### LIPH-LPA-LPAR axis enhanced PDAC glycolysis and viability by activating hypoxia and Hippo pathway

**Fig. 5:**
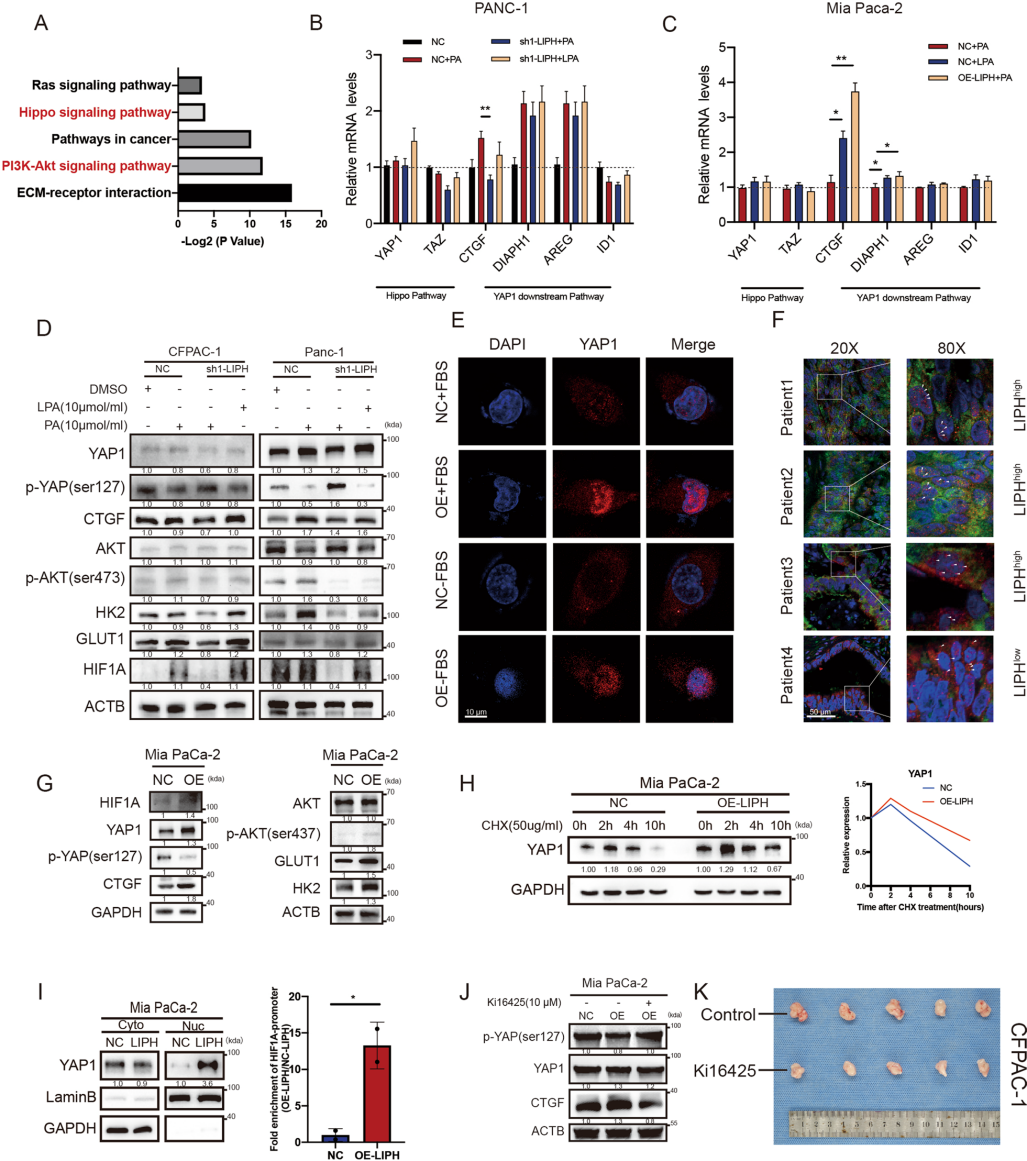
LIPH-LPA-LPAR axis enhanced PDAC glycolysis and viability by activating hypoxia and Hippo pathway.** A** KEGG pathway analysis showed enrichment of PI3K/AKT and Hippo pathway components in PANC-1 NC treated with PA (10 µM), compared to PANC-1 sh1-LIPH-transfected cells treated with PA (10 µM). **B** q-PCR showed that LIPH knockdown inhibited utilization of exogenous PA (10 µM) and downregulated CTGF expression at the mRNA level. **C** q-PCR showed that LIPH overexpression promoted utilization of exogenous PA (10 µM) as well as CTGF expression. **D** Immunoblot analysis of PDAC cells, with LIPH knockdown by sh1-LIPH or control, in the presence or absence of exogenous PA and LPA. **E** IF analysis of YAP1 localization of MIA PaCa-2 cells with LIPH overexpression by OE-LIPH vector and vector control in the presence or absence of FBS. **F** IF analysis of YAP1 and LIPH localization and expression level in PDAC patient tissues (red: YAP1, green: LIPH, blue: DAPI). **G** Immunoblot analysis of MIA PaCa-2 cells with LIPH overexpression by OE-LIPH or vector control. **H** Evaluation of the stability of YAP1 in MIA PaCa-2 cells stably transfected with OE-LIPH or vector control. Cells were treated with protein synthesis inhibitor cycloheximide (CHX; 50 μg/mL) and YAP1 abundance was examined by immunoblot of lysates of cells collected at the indicated times.** I** Abundance of YAP1 in cytosolic (Cyto) and nuclear (Nuc) fraction in MIA PaCa-2 cells stably transfected with OE-LIPH or vector control. Chip-qPCR analysis of abundance of YAP1 in HIF1A promoter in MIA PaCa-2 cells stably transfected with OE-LIPH or vector control. **J** Immunoblot analysis of MIA PaCa-2 cells with LIPH overexpression or vector control in the presence or absence of ki16425 (10 μM).** K** Subcutaneous tumor after the administration of ki16425 (10 mg/kg, qod) or corresponding solvent. Scale bar = 100 µm. *P < 0.05, **P < 0.01, ***P < 0.001 and ****P < 0.0001

Previous studies and our databases have confirmed that LPA-LPAR interaction is involved in PI3K/AKT signaling, hypoxia, and self-regeneration pathways, such as the Hippo pathway (Figs. 4A, 5A; Additional file 2: Fig. S2A) [17, 28]. Therefore, we first evaluated the mRNA expression of genes from the Hippo pathway and its downstream components. The expression of YAP/TAZ was relatively stable in each group, whereas a significant change of CTGF, a downstream gene of the Hippo pathway, was detected, suggesting higher utilization of exogenous PA cells in LIPH^high^ cells (Fig. 5B, C), which hinted a change in YAP/TAZ at the protein level, as well as an alteration in the localization of YAP/TAZ. To further understand the molecular effects arising from LIPH knockdown, two crucial transcription factors, YAP1 and HIF1A, were measured to explain the high ECAR levels and survival ability, even in a limited nutrient environment. The results showed that LIPH knockdown significantly promoted the phosphorylation of YAP1 at Ser^127^, a key phosphorylation site that mediates the degradation of YAP1 by ubiquitin [29] and inhibits the transcription of its downstream gene CTGF (Fig. 5D). In contrast, in PANC-1 and CFPAC-1 cells, LIPH knockdown inhibited the utilization of exogenous PA, limited the phosphorylation of AKT, reduced the expression of HIF1A, and further downregulated key downstream glycolysis genes such as HK2 and GLUT1 (Fig. 5D). Notably, treatment with exogenous LPA reversed the effects of LIPH knockdown (Fig. 5D). The subcellular localization of YAP1 determines its biological function [30]. LIPH overexpression promoted the utilization of PA and further enhanced the nuclear localization of YAP1 even in serum-deprived cultures (Fig. 5E). Patients with high LIPH expression also exhibited significant YAP1 nuclear localization, whereas YAP1 tended to localize in the cytoplasm in patients with low LIPH expression (Fig. 5F). Overexpression of LIPH in MIA PaCa-2 cells also reduced the phosphorylation of YAP1 at Ser^127^, promoted YAP1 nuclear localization, and conferred a high glycolysis phenotype to PDAC (Fig. 5G). These data indicate that LIPH enhances PDAC glycolysis and viability via the AKT/HIF1A pathway and inhibits the phosphorylation of YAP1.

To test whether the LIPH-LPA-LPAR axis affects the stability of YAP1, MIA PaCa-2 cells stably transfected with the control vector or overexpressing LIPH were treated with the protein synthesis inhibitor cycloheximide and YAP1 expression was monitored over time. Overexpression of LIPH stabilized YAP1 (Fig. 5H) and reduced proteasomal degradation of YAP1, which might be due to LPA-LPAR-mediated inhibition of LATS1/2 phosphorylation [17]. The subcellular localization of YAP1 is critical for its function, and phosphorylation of YAP1 promotes cytoplasmic retention of YAP1 and further degradation by ubiquitin. Cellular fractionation assays confirmed that the nuclear translocation of YAP1 was induced by LIPH overexpression (Fig. 5I). As an essential transcription factor, YAP1 sustains cellular metabolic reprogramming and self-regeneration by manipulating gene transcription [30]. GSEA showed a positive correlation between YAP1 and hypoxia and glycolysis pathways (Additional file 5: Fig. S5C). Given the significant enrichment in glycolysis and hypoxia pathway proteins based on our results and previous research [31–33], we focused on HIF1A, a vital transcriptional factor mediating hypoxia endurance, and abnormally activating glycolysis in tumors to explain the highly activated aerobic metabolism in LIPH^high^ tumors. LIPH overexpression effectively promoted YAP1 nuclear localization as well as its combination with the HIF1A transcription start site (Fig. 5I). Finally, we used ki16425, an LPAR1/3 inhibitor, to explore the role of LIPH-LPA-LPAR axis in PDAC. Using ki16425 effectively promoted the phosphorylation of YAP1, inhibited the expression of its downstream gene, CTGF (Fig. 5J), and restrained the growth of subcutaneous tumors (Fig. 5K; Additional file 5: Fig. S5D). Together, these data indicate that nuclear localization of HIF1A and YAP1 is responsible for LIPH-mediated tumorigenesis and abnormal glycolysis.

### The lipase LIPH supported ALDOA stability via reducing UBE2O combination

**Fig. 6:**
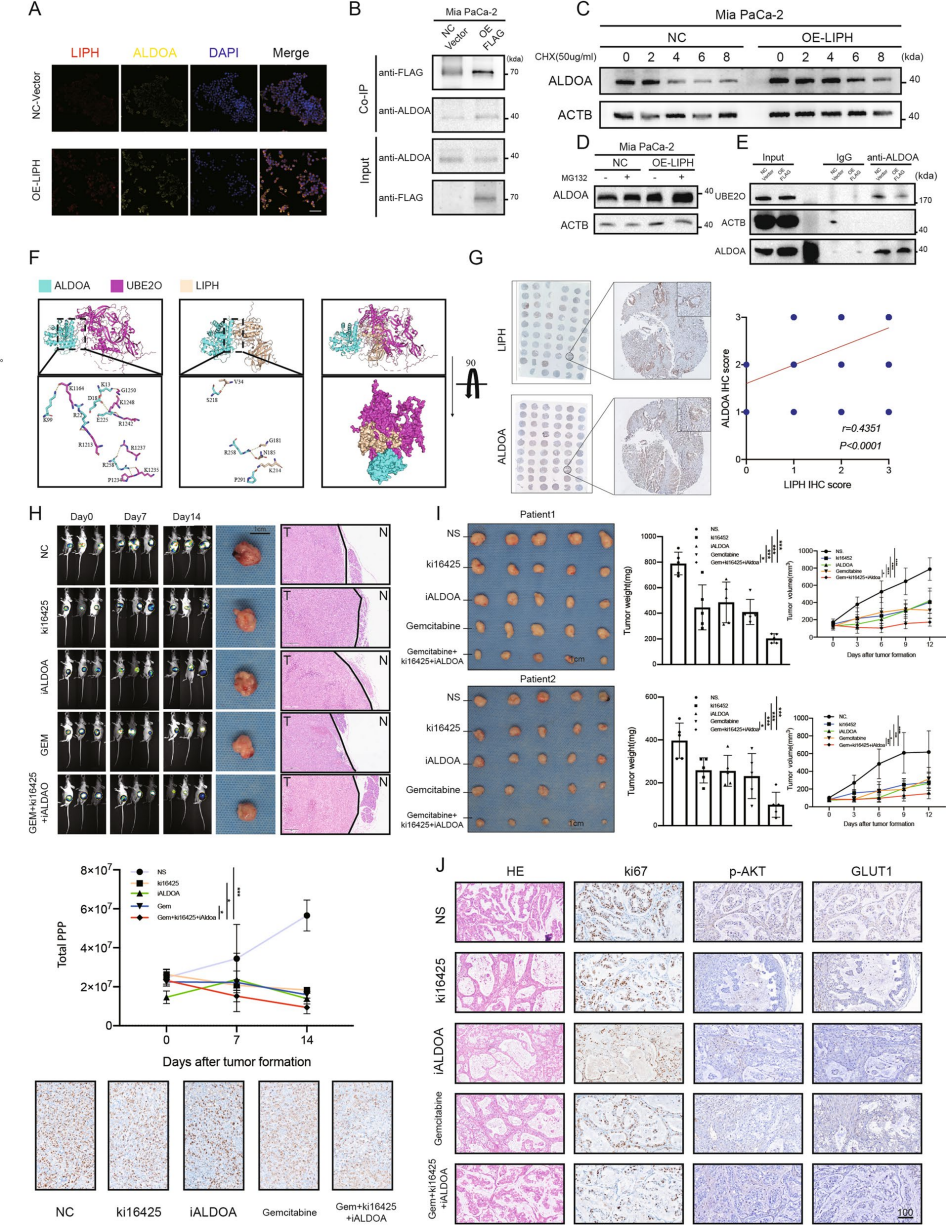
The Lipase LIPH supported ALDOA stability by reducing its combination with UBE2O.** A** Immunofluorescence staining of LIPH and ALDOA in MIA PaCa-2 cells transfected with the NC- and OE-LIPH vectors **B** Co-IP verified the interaction between LIPH and ALDOA. **C** Evaluation of ALDOA stability in MIA PaCa-2 cells stably transfected with OE-LIPH or control vectors. Cells were treated with protein synthesis inhibitor cycloheximide (CHX; 50 μg/mL) and ALDOA abundance was examined by immunoblot of lysates of cells collected at the indicated times. **D** ALDOA protein levels in MIA PaCa-2 cells overexpressing LIPH were treated with MG132 (20 μM) for 12 h. **E** Co-IP verified the interaction between UBE2O and ALDOA in MIA PaCa-2 cells transfected with the NC or OE-LIPH vectors. **F** The binding sites between ALDOA and UBE2O were modeled using molecular docking. In terms of space, steric hindrance after the combination of LIPH and ALDOA prevents UBE2O from approaching ALDOA (the surface diagram after SuperPose shows that the two have a large overlapping area, indicating that their spatial positions, when combined with ALDOA, are highly similar; that is, under the same space-time conditions, only one of the LIPH or UBE2O molecules can bind to ALDOA. **G** Standard IHC images and respective sample counts of ALDOA expression in 99 pancreatic ductal adenocarcinoma and paired adjacent normal tissues. **H** The orthotopic xenograft model, showing bioluminescence data at the indicated time points, and corresponding H&E staining and ki67 expression. **I** Two patient-derived xenograft (PDX) models were constructed, volume was measured at the indicated time points, and tumor weight was measured at the end of the experiment. **J** H&E stained PDX tissue, and IHC staining of ki67, p-AKT, GLUT1. Scale bar = 100 µm. *P < 0.05, **P < 0.01, ***P < 0.001 and ****P < 0.0001

In addition to localization on the membrane, a large amount of endogenous LIPH was found to be universally distributed in the cytosol (Fig. 1E), which has not been discussed in previous publications, and neither has its potential role in glycolysis. To explore this, LC–MS was performed for the products of Co-IP with the FLAG antibody. Various glycolytic enzymes, including ENO1, ALDOA, LDHB, and G6PD showed combination with LIPH (Additional file 6: Fig. S6A; Fig. 6B). ALDOA, a hub enzyme directly connecting lipid and glucose metabolism, which has a high overlap with LIPH in biological functions, was chosen for further analysis [19]. Multi-IF showed that ALDOA and LIPH were co-expressed in the cytosol and that the expression of ALDOA was higher in cells transfected with the OE-LIPH plasmid (Fig. 6A). More ALDOA protein was immunoprecipitated with anti-FLAG in LIPH-overexpressing cells in the co-IP analysis (Fig. 6B), and overexpression of LIPH also elevated ALDOA levels in the cytosol (Fig. 6E). In addition to a slight change in ALDOA mRNA levels (Fig. 3B), which might contribute to its expression in the cytosol, LIPH overexpression significantly enhanced the stability of ALDOA (Fig. 6C), with less degradation through the proteasome pathway (Fig. 6D). The potential ubiquitin sites were predicted using iUUCD (Additional file 6: Fig. S6C). Screening of proteins that interacted with LIPH in MIA PaCa-2 cells transfected with the NC-vector and OE-LIPH revealed that UBE2O, an E2/E3 hybrid ubiquitin-protein ligase that displays both E2 and E3 ligase activities and mediates mono-ubiquitination of target proteins, may be responsible for the elevated expression of ALDOA (Fig. 6E; Additional file 6: Fig. S6B). We hypothesized that the combination of LIPH and ALDOA would reduce the access of UBE2O to potential ubiquitin sites in ALDOA, such as K13, thereby maintaining a high rate of glycolysis in LIPH^high^ tumors (Fig. 6F). The protein levels of LIPH and ALDOA in the tumor tissues were highly positively correlated (Fig. 6G). Abnormally high ALDOA expression predicted an unfavorable prognosis (Additional file 6: Fig. S6D). Collectively, by competing with UBE2O, LIPH maintained the stability of ALDOA, thereby maintaining glycolysis.

### Combination therapy effectively inhibited PDAC progression

Given the lack of direct and effective LIPH inhibitors, a three-drug combination therapy (ki16425/aldometanib/gemcitabine, KAG) was administered to restrict tumor glycolysis and progression. The bioluminescence data revealed that KAG-treated mice showed a slower rate of tumor growth than the control mice and the single-drug group (Fig. 6H). To further test this drug combination, we generated a patient-derived murine xenograft tumor model. IHC staining of PDX xenograft tumor samples confirmed the pathological diagnosis and showed high LIPH expression in both (Additional file 6: Fig. S6F). KAG contributed to a remarkable decrease in tumor growth (Fig. 6I) with limited side effects (Additional file 6: Fig. S6E). The tumors were resected, and histological sections were prepared for further investigation. As expected, significant inhibition of Ki67, p-AKT, and GLUT1 expression was observed in the KAG group (Fig. 6J; Additional file 6: Fig. S6G). The results show KAG effectively restrained tumor progression in vivo and combination therapy with gemcitabine may provide additional benefits.

### The lipase LIPH is activated by the oncogenic GTPase KRAS

Since mutant KRAS is a critical mediator of pancreatic cancer metabolism [5], we evaluated the association between KRAS mutations and LIPH expression. KRAS^mut^ tumors were highly positive for LIPH expression in the TCGA, MTAB-6134, and Ruijin cohorts (Additional file 6: Fig. S6H). A strong positive correlation was observed between LIPH and KRAS expression (Additional file 6: Fig. S6I). Cell line analysis showed that both non-neoplastic HPNE harbored wild-type KRAS and showed lower levels of LIPH expression than PANC-1, CFPAC-1, ASPC-1, 8988s, and 8988t cells, which have an activating mutation in KRAS (Fig. 2A) [34]. Interestingly, LIPH levels were not simply associated with KRAS mutation status, as Mia PaCa-2 cells harboring KRAS mutations expressed lower levels of LIPH than the wild-type KRAS cell line HPNE, which may be due to its semi-adherent phenotype. Mouse-derived cell lines [35] with KRAS^mut^ exhibit higher LIPH expression (Additional file 6: Fig. S6J). We also examined LIPH expression in pancreatic tissues of mice expressing KRAS^G12D^. We confirmed that LIPH expression was higher than that in wild-type mice (Additional file 6: Fig. S6K). We investigated the correlation between LIPH and oncogenic KRAS using a doxycycline-inducible cell culture system. BxPC-3^wild type KRAS^ were stably transfected with the doxycycline-inducible lentiviral KRAS^G12D^ construct. Following the removal of doxycycline, the cells reverted to wild-type KRAS, as confirmed by the downstream ERK pathway. Reversion to wild-type KRAS gradually decreased the LIPH expression (Additional file 5: Fig. S5L). KRAS signaling plays a pivotal role in biological processes through several downstream pathways. To further confirm which signaling pathway mediates the high expression of LIPH, the MEK inhibitor U0126 was utilized. MEK inhibition significantly downregulated the expression of LIPH in the PANC-1 and CFPAC-1 cell lines (Additional file 6: Fig. S6M), indicating that KRAS-mediated LIPH upregulation occurred through the MEK/ERK pathway. Taken together, KRAS^mut^ elevates LIPH expression and promotes metabolic reprogramming in PDAC cells.

## Discussion

**Fig. 7:**
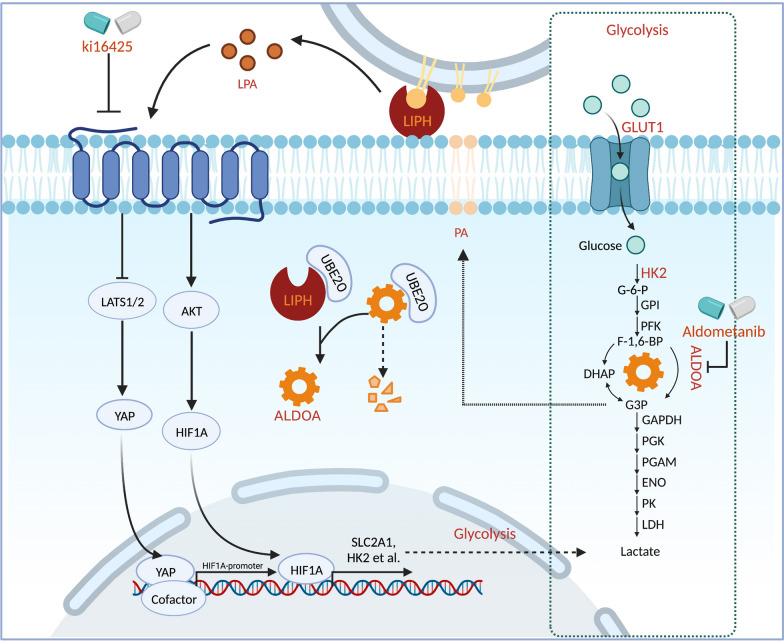
Proposed model demonstrating that expression of LIPH promotes PC tumorigenesis via PA/LIPH/LPA axis and maintaining ALDOA stability

The TME supports the progression of PDAC by facilitating cancer cell proliferation, immune evasion, and metabolic reprogramming. SPs directly bridge cancer cells and the surrounding microenvironment, mediating aberrant proliferation rates even with limited nutrient supply. In this study, we identified LIPH as an actionable target for PDAC treatment. This study demonstrated that LIPH expression is upregulated and predicts poor overall survival in PDAC. LIPH catalyzes the conversion of PA to LPA and facilitates cancer cell proliferation by enhancing YAP1 nuclear localization, stimulating the PI3K/AKT/HIF1A pathway, and maintaining ALDOA stability, ultimately resulting in aberrant glycolysis and tumorigenesis (Fig. 7).

Previous reports have validated serum LPA as a potential diagnostic biomarker and a potent extracellular signaling lipid in patients with pancreatic cancer [10, 36]. Although blood circulation is a tremendous LPA reservoir, PDAC may benefit limited by blood LPA due to poor vascularization [37]. In this study, we found that LIPH, a membrane-conjugated enzyme that is highly expressed in PDAC cells, can convert extracellular PA to LPA and support cell proliferation by reprogramming cancer cell metabolism. Transcriptomic and metabolic analyses indicated that the growth-promoting role of LIPH is highly dependent on glycolysis. Furthermore, the mechanisms of metabolic reprogramming and tumorigenesis induced by LIPH are largely ascribed to HIF1A-mediated transcript activation, YAP1 nuclear localization, and ALDOA stabilization. Consistent with our research, a previous study on ovarian cancer indicated that extracellular LPA could reprogram cancer cell metabolism via an HIF1A-mediated pseudo-hypoxic response, indicating that HIF1A regulation by LPA might be a common mechanism in tumorigenesis [38]. YAP1 nuclear localization also influenced glycolysis by stimulating GLUT3 expression, or by indirectly regulating the transcription of HK2 and PFKB3 by the long non-coding RNA BCAR4 [39]. Although we do not discuss the relationship between YAP1 and glycolysis in detail here, our findings and recent studies suggest that YAP1 promotes the transcription of HIF1A in cancer, indicating that LPA could affect tumor glycolysis through multiple upstream pathways [32].

Pancreatic cancer cells can access bioactive lipids (such as PA and LPA) from various sources. Obesity, which systematically elevates serum lipid levels, is associated with pancreatic cancer with poor prognosis, and the possibility of distant metastasis [40–42]. Furthermore, feeding a high-fat, high-calorie diet to LSL-KRAS^G12D^ (KC) mice carrying a pancreas-specific oncogenic KRAS mutation prominently increased the incidence of PDAC [43]. Additionally, PDAC cells exhibit elevated levels of lipid synthesis and accumulate stored lipids in response to hypoxia [44]. The PA-generating enzyme, LPAAT-β, plays an essential role in triglyceride synthesis, which further regulates mTOR signaling to support PDAC progression [45–47]. As a hub enzyme connecting glucose and lipid metabolism, ALDOA catalyzes the reversible conversion of fructose 1,6-bisphosphate to glyceraldehyde-3-phosphate and dihydroxyacetone phosphate, which can be subsequently converted to PA [19]. Our data suggested that LIPH supports ALDOA stability, promoting glycolysis and maintaining the PA reservoir. Besides, because the k_d_ for LPAR is 10–40 nmol/L, we considered that even low LPA concentrations synthesized by LIPH might have potent biological effects [48].

Activating mutations in KRAS drive more than 90% of pancreatic cancers and are thought to initiate a shift towards glycolysis to sustain rapid energy demand in the malignant tumor [49]. In this study, we hypothesized that aberrant activation of KRAS drives the high expression of LIPH, subsequent YAP1 nuclear localization, and HIF1A-mediated aerobic glycolysis. Continuous efforts have been made to target KRAS [50], HIF1A [51], and YAP1 [52] because of their roles in tumor initiation. However, considering their roles in normal development, potential inhibitors should be carefully considered before undertaking clinical trials. Thus, instead of direct inhibition, targeting LIPH in pancreatic cancer cells might be a possible therapeutic avenue. However, to date, there is a lack of effective drugs that directly target LIPH. In the present study, we designed a new drug combination for the treatment of PDAC. KAG demonstrated effective tumor constraint with no significant influence on the mouse weight. Ki16425 effectively inhibited LPAR1/3, whereas aldometanib specifically inhibited lysosome-pool aldolase without impairing glycolysis or causing discernible side effects. In addition, aldometanib improves glucose homeostasis, alleviates fatty liver and NASH, and extends the lifespan and health span of nematodes and mice, which might benefit patients with PDAC because most patients with PDAC suffer from varying degrees of diabetes [20, 53].

One limitation of this study is that we do not discuss the formation and progression of cancer cells without LIPH expression, as well as corresponding target therapy. Thus, further sc-RNA seq may be needed to reveal these cells in PDAC relapse and chemotherapy resistance. Besides, we do not clarify the crosstalk between PI3K/AKT/HIFα and the Hippo pathway, which may contain potential intervention methods.

### Supplementary Information

**Additional file 1: Figure S1.** LIPH was significantly upregulated in PDAC and was related to later pathological stage and poor prognosis.** A** GSE15471, GSE16515 and GSE28735 were used to screen for SPs that were expressed at significantly higher levels in PDAC tissues than adjacent areas (Cross1, 108 genes). **B** GSE71729 was used to filter SPs (Cross1) specifically expressed (17 genes) in PDAC rather than normal tissues to minimize the drug toxicity of potential target treatment. **C** The t-SNE plot (CRA001160) presented all sequenced cells based on cell type. The feature plot shows LIPH expression in t-SNE map. **D&E** Trajectory plots showed LIPH, CCNB2, and MUC1 gradually elevated along pseudo-time. CCNB2 is a known proliferation-associated gene. MUC1 is a gene that was used to distinguish the abnormal and malignant gene expression profiles of ductal cells [54]. **F** LIPH expression of 29 tumor cell lines in CCLE datasets. **G** LIPH expression in GTEx and TCGA datasets. **H** LIPH expression in 99 pairs of PDAC and adjacent tissues from GSE15471, GSE16515 and GSE62452. **I** Disease-free survival of LIPH based on Ruijin cohort. **J** Multivariate Cox regression analysis based on Ruijin cohort. *P < 0.05, **P < 0.01, ***P < 0.001 and ****P < 0.0001.

**Additional file 2: Figure S2** LIPH promoted proliferation and inhibited apoptosis of pancreatic cancer cells in vitro*. A* GSVA analysis of the Ruijin cohort showed glycolysis, hypoxia and Hippo pathways were enriched in LIPH^high^ tumor tissues. **B** The mRNA and protein expression of LIPH in LIPH-overexpressing MIA PaCa-2 cells. **C** The CCK8 assay showed that LIPH overexpression in MIA PaCa-2 resulted in elevated proliferation ability. The colony formation ability was elevated by LIPH overexpression. **D** Flow cytometric apoptosis assay and BAX protein were used to detect the apoptosis level associated with LIPH overexpression. **E** Survival of CFPAC-1 cells (vector and sh1-LIPH) and MIA PaCa-2 cells (vector and OE-LIPH) treated with gradient gemcitabine. **F** MIA PaCa-2 cells were cultured for one day in 0.5% FAF-BSA complete medium (starvation), and then exogenous PA (10 µM) or LPA (10 µM) was added to the culture, followed by CCK8 assays at 72 h to detect cell proliferation ability.** G** Cells were cultured for one day in 0.5% FAF-BSA complete medium (starvation), and then exogenous PA (10 µM) or LPA (10 µM) was added to the culture, followed by crystal violet staining to evaluate colony formation ability. *P < 0.05, **P < 0.01, ***P < 0.001 and ****P < 0.0001.

**Additional file 3: Figure S3** LIPH Knockdown inhibited xenograft tumor growth.** A** LIPH expression in xenograft tumor. **B** Representative images of IHC staining of HK2, GLUT1, CTGF, Ki67. **C** The intensity of HK2, GLUT1 and CTGF. Scale bar = 100 µm. *P < 0.05, **P < 0.01, ***P < 0.001 and ****P < 0.0001.

**Additional file 4: Figure S4** LPA produced by LIPH promoted PDAC progression through enhancing glycolysis.** A** GSEA using hallmark gene sets was performed to compare the LIPH^high^ and LIPH^low^ groups in GSE15471, TCGA datasets, and GSE28735. NES, normalized enrichment score. **B** Acidification rates were calculated by Seahorse analysis (PANC-1 left, CFPAC-1 right). **C** ECAR and acidification rates detected by Seahorse analyzer in vector and OE-LIPH transfected MIA PaCa-2. Cells were seeded and cultured for one day in 0.5% FAF-BSA complete medium (starvation), and then exogenous PA (10 µM) or LPA (10 µM) was added to cultured cell before analysis (Glc: Glucose; O: oligomycin; 2-DG: 2-deoxyglucose). **D** Oxygen consumption rate (OCR) detected by Seahorse analyzer (O: oligomycin; F: FCCP; R&A: rotenone & actinomycin A). *P < 0.05, **P < 0.01, ***P < 0.001 and ****P < 0.0001.

**Additional file 5: Figure S5** LIPH-LPA-LPAR axis enhanced PDAC glycolysis and viability by activating hypoxia and Hippo pathways.** A** Positive association between YAP1 and LIPH in TCGA datasets. **B** q-PCR showed that LIPH knockdown inhibited utilization of exogenous PA and downregulated CTGF expression at the mRNA level. **C** GSEA using hallmark gene sets was performed to compare the YAP1^high^ and YAP1^low^ groups in Ruijin cohort datasets (Cutoff = median value). NES, normalized enrichment score. **D** Weight of subcutaneous tumor after administration of ki16425 (10 mg/kg, qod) or corresponding solvent. *P < 0.05, **P < 0.01, ***P < 0.001 and ****P < 0.0001.

**Additional file 6: Figure S6** The Lipase LIPH supported ALDOA stability via reducing combination with UBE2O.** A** Silver staining of LIPH-associated proteins. **B** The abundance of glycolysis proteins and ubiquitin protein measured by LC–MS. **C** Potential ubiquitin site provided by iUUCD (http://iuucd.biocuckoo.org/). **D** Standard IHC images and respective sample count of ALDOA expression in 99 pancreatic ductal adenocarcinoma and paired adjacent normal tissues, and overall survival. **E** Mice weights fluctuated during treatment. Geometric mean, 95% Cl and two-way ANOVA test was used for comparison. **F** Representative images of LIPH and ALDOA in two PDX(F1). **G** representative images of H&E, ki67 and GLUT1 staining in the PDX derived from patient 1. Scale bar = 100 µm. **H** The expression of LIPH in PDAC tissue with or without KRAS mutation in TCGA, MTAB-6134 and Ruijin cohort. **I** Positive association between KRAS and LIPH in TCGA datasets. **J** Immunoblot analysis of LIPH expression in Panc02 and KPC1199 cells. **K** Expression of LIPH in the pancreas of wild type C57BL/6 and KrasG12D/ + /Trp-53R172H/ + /Pdx1-Cre (KPC) mice. **L** Immunoblot analysis of LIPH, ERK and p-ERK in BxPC-3^KRASG12D−dox^. **M** The expression of LIPH in CFPAC-1 and PANC-1 cells treated with U0126 (10 mmol/L) at indicated times. *P < 0.05, **P < 0.01, ***P < 0.001 and ****P < 0.0001.

**Additional file 7: Table S1**. The information of the 10 genes in Veen.

**Additional file 8.** Extended information.

## Data Availability

The authors declare that the data supporting the findings of this study are available within the paper.

## Abbreviations

2-DG: 2-Deoxyglucose
ATP: Adenosine triphosphate
ALDOA: Aldolase A
BSA: Bovine serum albumin
CCK-8: Cell Counting Kit-8
ChIP: Chromatin immunoprecipitation
DMSO: Dimethyl sulfoxide
ECAR: Extracellular acid rate
FBS: Fetal bovine serum
FAF-BSA: Fatty acid free-bovine serum albumin
GEO: Gene Expression Omnibus
GSVA: Gene set variation analysis
GEM: Gemcitabine
GTEx: The Genotype-Tissue Expression
IF: Immunofluorescence
IHC: Immunohistochemistry
KPC mice: KrasG12D/+/Trp53R172H/+/Pdx1-Cre mice
LIPH: Lipase H
LPA: Lysophosphatidic acid
LC–MS: Liquid chromatography–mass spectrometry
NC: Negative control
OE: Overexpression
OCR: Oxygen consumption rate
PA: Phosphatidic acid
PDAC: Pancreatic ductal adenocarcinoma
PET/CT: Positron emission tomography/computed tomography
SPs: Cell-surface proteins
SUVMax: Maximum standardized uptake value
TCGA: The Cancer Genome Atlas
TMA: Tissue microarray
TUNEL: Terminal deoxynucleotidyl transferase–mediated deoxyuridine triphosphate nick-end labelling
WT: Wild type
