# Supplementary figures and images for: LIPH contributes to glycolytic phenotype in pancreatic ductal adenocarcinoma by activating LPA/LPAR axis and maintaining ALDOA stability
Source: J Transl Med. 2023 Nov 21;21:838. doi: 10.1186/s12967-023-04702-6 (PMC10664664; doi:10.1186/s12967-023-04702-6)

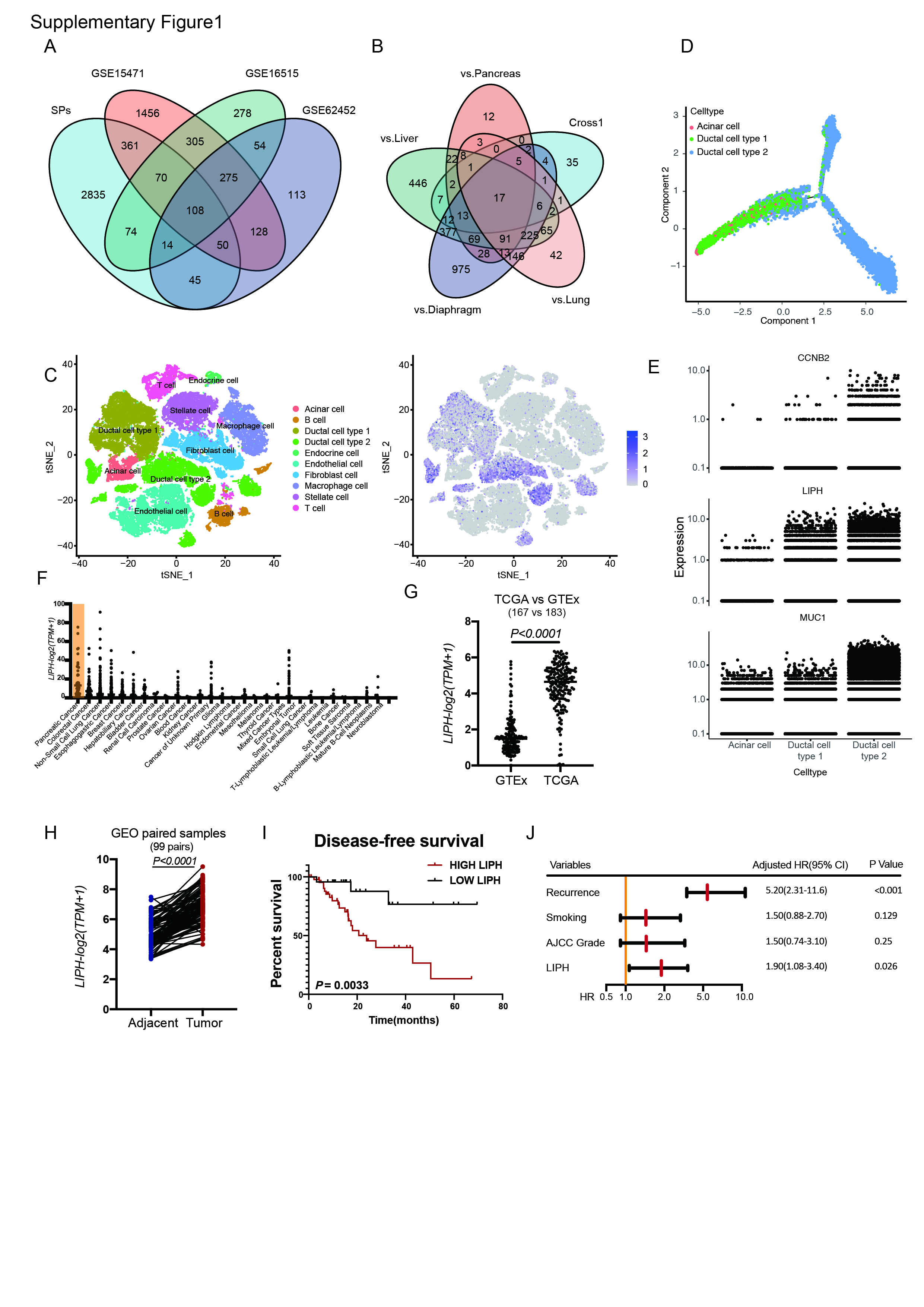

Supplement: Supplementary file 1 — Additional file 1: Figure S1. LIPH was significantly upregulated in PDAC and was related to later pathological stage and poor prognosis. A GSE15471, GSE16515 and GSE28735 were used to screen for SPs that were expressed at significantly higher levels in PDAC tissues than adjacent areas (Cross1, 108 genes). B GSE71729 was used to filter SPs (Cross1) specifically expressed (17 genes) in PDAC rather than normal tissues to minimize the drug toxicity of potential target treatment. C The t-SNE plot (CRA001160) presented all sequenced cells based on cell type. The feature plot shows LIPH expression in t-SNE map. D&E Trajectory plots showed LIPH, CCNB2, and MUC1 gradually elevated along pseudo-time. CCNB2 is a known proliferation-associated gene. MUC1 is a gene that was used to distinguish the abnormal and malignant gene expression profiles of ductal cells [54]. F LIPH expression of 29 tumor cell lines in CCLE datasets. G LIPH expression in GTEx and TCGA datasets. H LIPH expression in 99 pairs of PDAC and adjacent tissues from GSE15471, GSE16515 and GSE62452. I Disease-free survival of LIPH based on Ruijin cohort. J Multivariate Cox regression analysis based on Ruijin cohort. *P < 0.05, **P < 0.01, ***P < 0.001 and ****P < 0.0001. [file 12967_2023_4702_MOESM1_ESM.jpg]

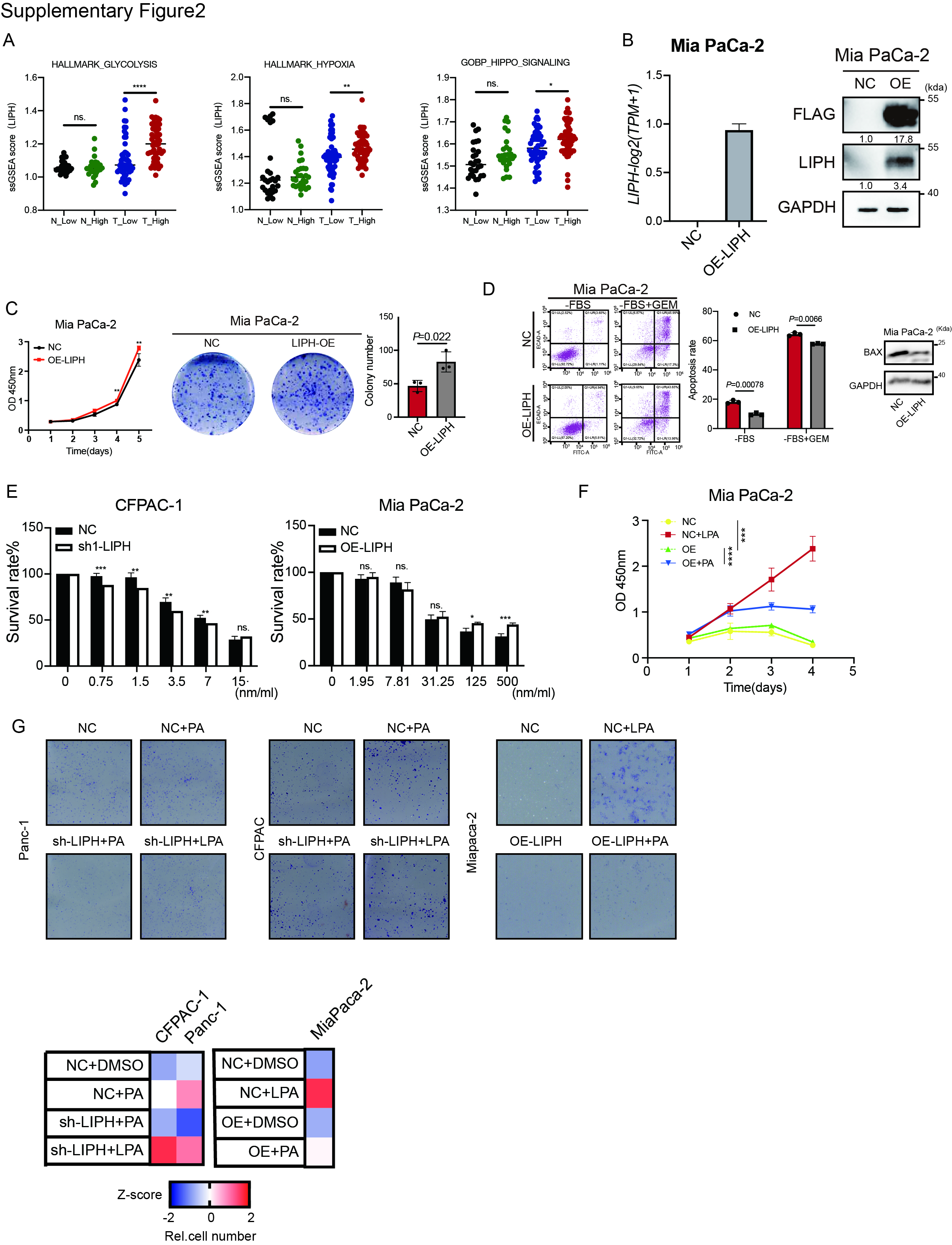

Supplement: Supplementary file 2 — Additional file 2: Figure S2 LIPH promoted proliferation and inhibited apoptosis of pancreatic cancer cells in vitro. A GSVA analysis of the Ruijin cohort showed glycolysis, hypoxia and Hippo pathways were enriched in LIPHhigh tumor tissues. B The mRNA and protein expression of LIPH in LIPH-overexpressing MIA PaCa-2 cells. C The CCK8 assay showed that LIPH overexpression in MIA PaCa-2 resulted in elevated proliferation ability. The colony formation ability was elevated by LIPH overexpression. D Flow cytometric apoptosis assay and BAX protein were used to detect the apoptosis level associated with LIPH overexpression. E Survival of CFPAC-1 cells (vector and sh1-LIPH) and MIA PaCa-2 cells (vector and OE-LIPH) treated with gradient gemcitabine. F MIA PaCa-2 cells were cultured for one day in 0.5% FAF-BSA complete medium (starvation), and then exogenous PA (10 µM) or LPA (10 µM) was added to the culture, followed by CCK8 assays at 72 h to detect cell proliferation ability. G Cells were cultured for one day in 0.5% FAF-BSA complete medium (starvation), and then exogenous PA (10 µM) or LPA (10 µM) was added to the culture, followed by crystal violet staining to evaluate colony formation ability. *P < 0.05, **P < 0.01, ***P < 0.001 and ****P < 0.0001. [file 12967_2023_4702_MOESM2_ESM.jpg]

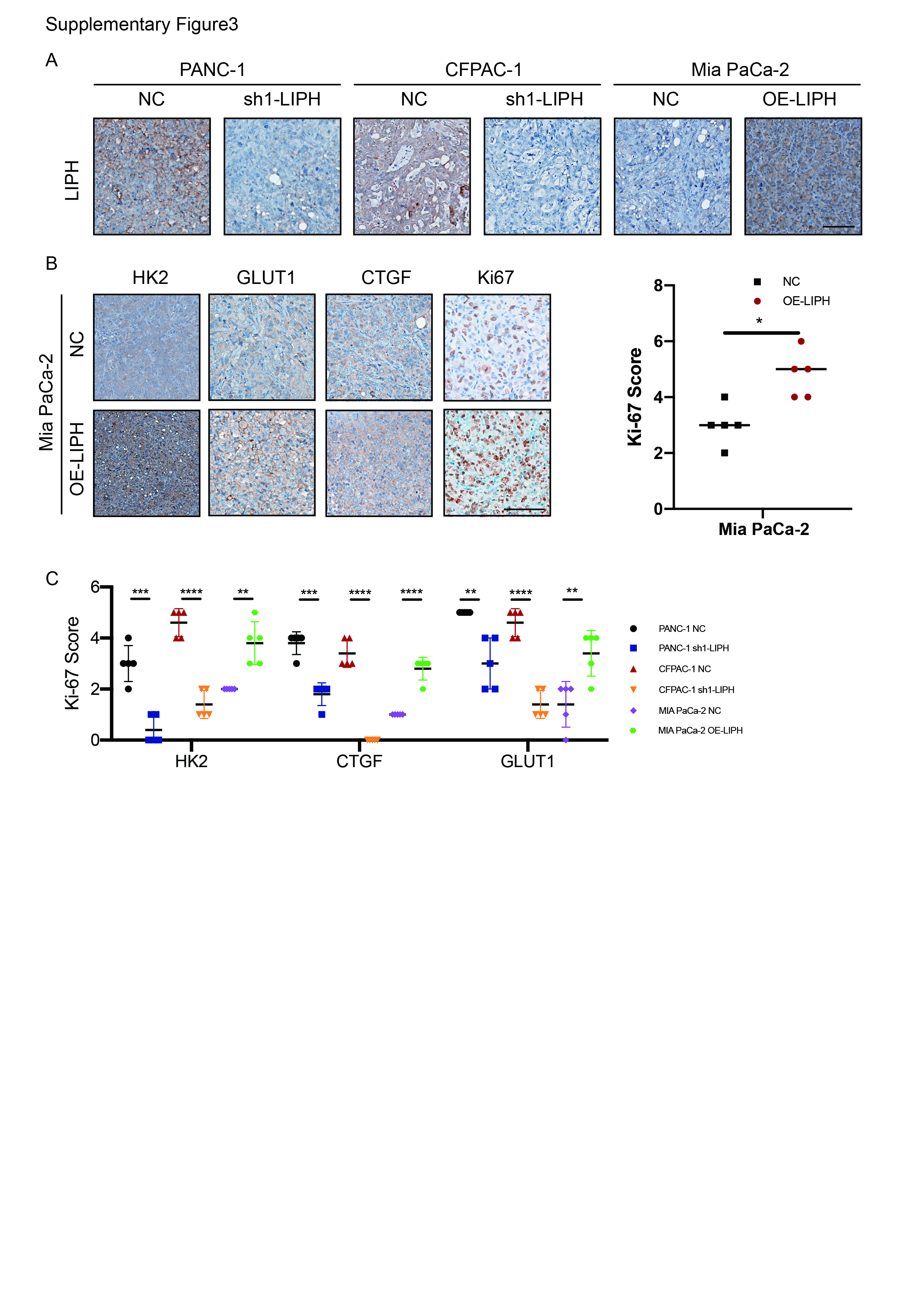

Supplement: Supplementary file 3 — Additional file 3: Figure S3 LIPH Knockdown inhibited xenograft tumor growth. A LIPH expression in xenograft tumor. B Representative images of IHC staining of HK2, GLUT1, CTGF, Ki67. C The intensity of HK2, GLUT1 and CTGF. Scale bar = 100 µm. *P < 0.05, **P < 0.01, ***P < 0.001 and ****P < 0.0001. [file 12967_2023_4702_MOESM3_ESM.jpg]

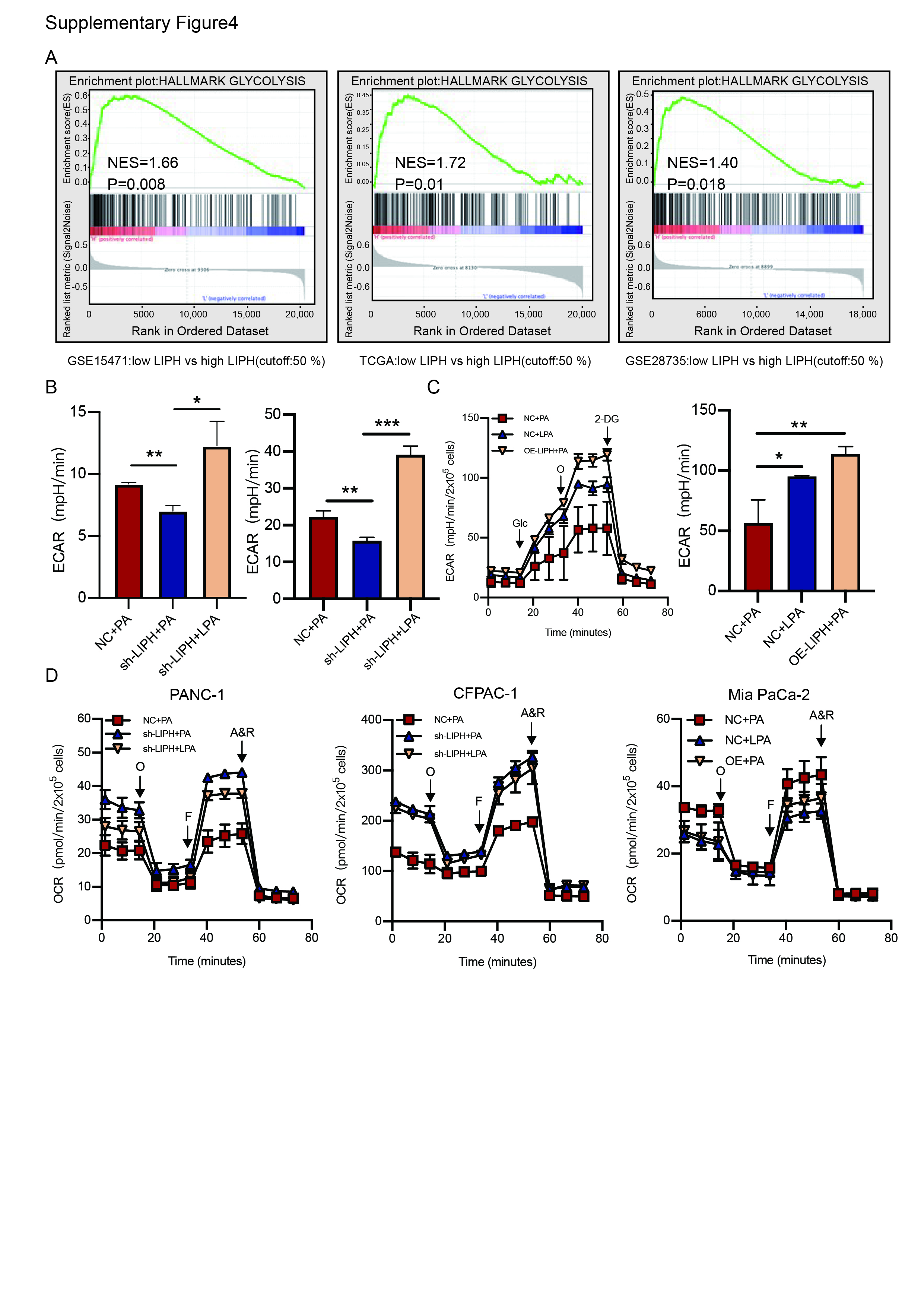

Supplement: Supplementary file 4 — Additional file 4: Figure S4 LPA produced by LIPH promoted PDAC progression through enhancing glycolysis. A GSEA using hallmark gene sets was performed to compare the LIPHhigh and LIPHlow groups in GSE15471, TCGA datasets, and GSE28735. NES, normalized enrichment score. B Acidification rates were calculated by Seahorse analysis (PANC-1 left, CFPAC-1 right). C ECAR and acidification rates detected by Seahorse analyzer in vector and OE-LIPH transfected MIA PaCa-2. Cells were seeded and cultured for one day in 0.5% FAF-BSA complete medium (starvation), and then exogenous PA (10 µM) or LPA (10 µM) was added to cultured cell before analysis (Glc: Glucose; O: oligomycin; 2-DG: 2-deoxyglucose). D Oxygen consumption rate (OCR) detected by Seahorse analyzer (O: oligomycin; F: FCCP; R&A: rotenone & actinomycin A). *P < 0.05, **P < 0.01, ***P < 0.001 and ****P < 0.0001. [file 12967_2023_4702_MOESM4_ESM.jpg]

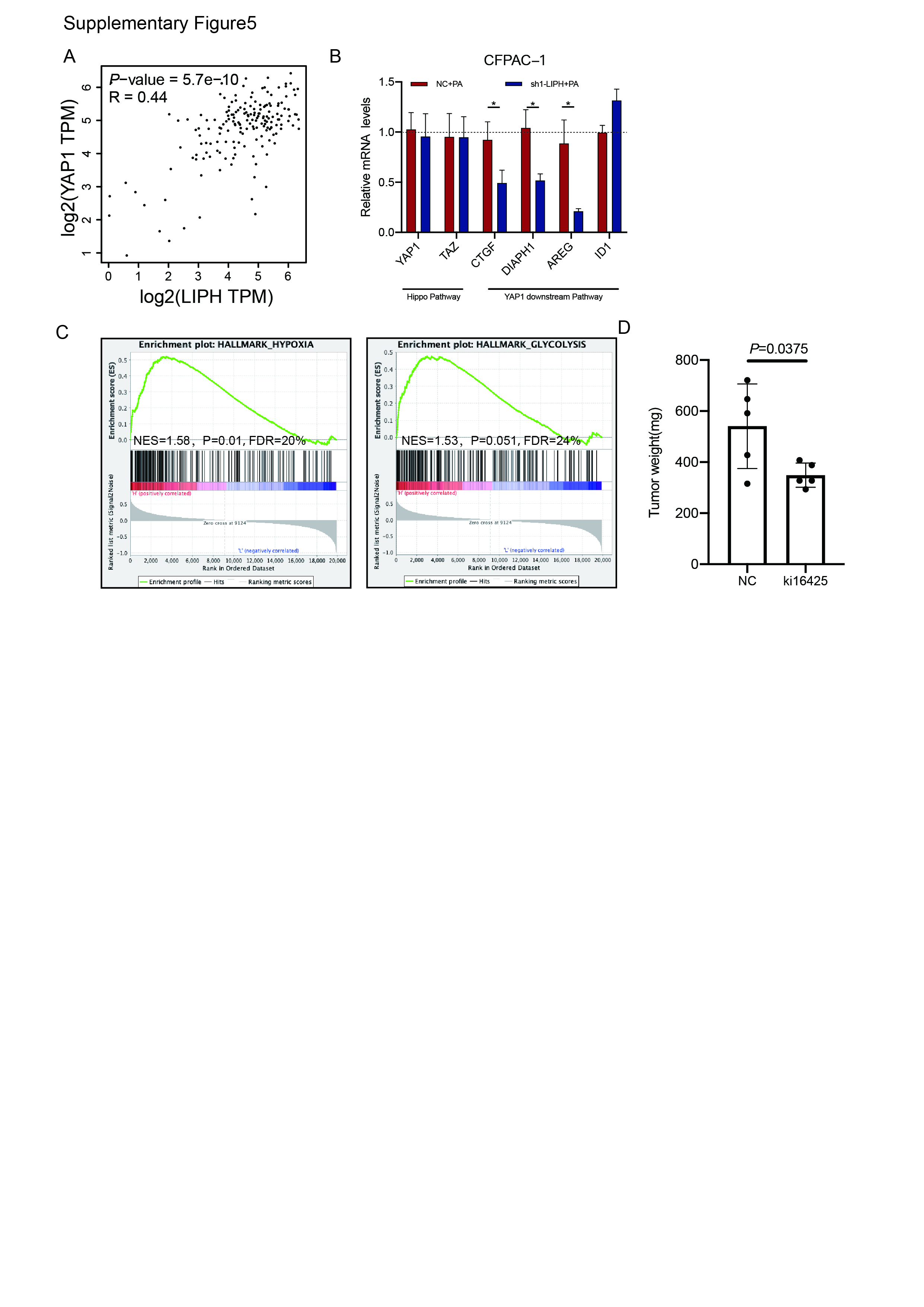

Supplement: Supplementary file 5 — Additional file 5: Figure S5 LIPH-LPA-LPAR axis enhanced PDAC glycolysis and viability by activating hypoxia and Hippo pathways. A Positive association between YAP1 and LIPH in TCGA datasets. B q-PCR showed that LIPH knockdown inhibited utilization of exogenous PA and downregulated CTGF expression at the mRNA level. C GSEA using hallmark gene sets was performed to compare the YAP1high and YAP1low groups in Ruijin cohort datasets (Cutoff = median value). NES, normalized enrichment score. D Weight of subcutaneous tumor after administration of ki16425 (10 mg/kg, qod) or corresponding solvent. *P < 0.05, **P < 0.01, ***P < 0.001 and ****P < 0.0001. [file 12967_2023_4702_MOESM5_ESM.jpg]

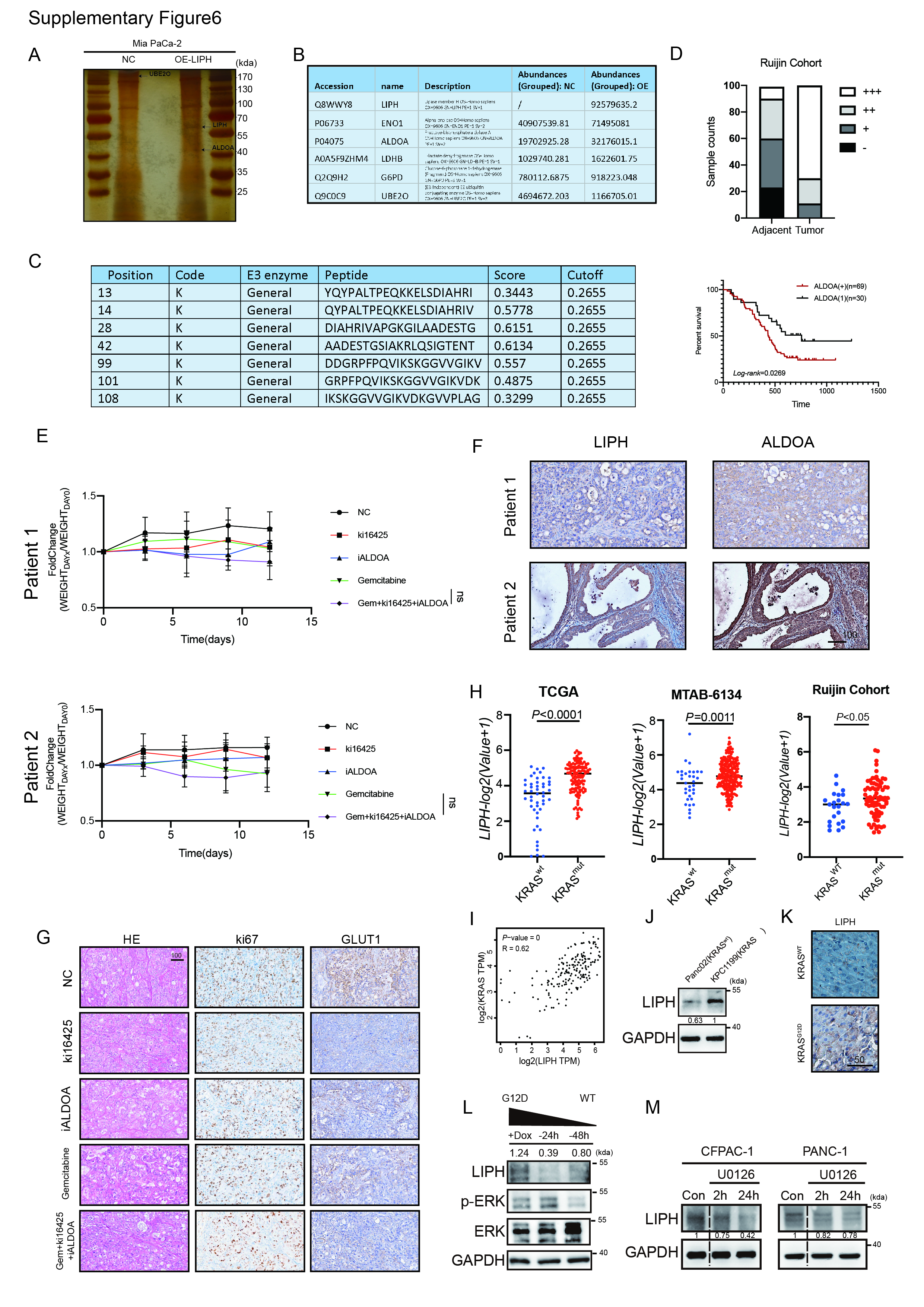

Supplement: Supplementary file 6 — Additional file 6: Figure S6 The Lipase LIPH supported ALDOA stability via reducing combination with UBE2O. A Silver staining of LIPH-associated proteins. B The abundance of glycolysis proteins and ubiquitin protein measured by LC–MS. C Potential ubiquitin site provided by iUUCD (http://iuucd.biocuckoo.org/). D Standard IHC images and respective sample count of ALDOA expression in 99 pancreatic ductal adenocarcinoma and paired adjacent normal tissues, and overall survival. E Mice weights fluctuated during treatment. Geometric mean, 95% Cl and two-way ANOVA test was used for comparison. F Representative images of LIPH and ALDOA in two PDX(F1). G representative images of H&E, ki67 and GLUT1 staining in the PDX derived from patient 1. Scale bar = 100 µm. H The expression of LIPH in PDAC tissue with or without KRAS mutation in TCGA, MTAB-6134 and Ruijin cohort. I Positive association between KRAS and LIPH in TCGA datasets. J Immunoblot analysis of LIPH expression in Panc02 and KPC1199 cells. K Expression of LIPH in the pancreas of wild type C57BL/6 and KrasG12D/ + /Trp-53R172H/ + /Pdx1-Cre (KPC) mice. L Immunoblot analysis of LIPH, ERK and p-ERK in BxPC-3KRASG12D−dox. M The expression of LIPH in CFPAC-1 and PANC-1 cells treated with U0126 (10 mmol/L) at indicated times. *P < 0.05, **P < 0.01, ***P < 0.001 and ****P < 0.0001. [file 12967_2023_4702_MOESM6_ESM.jpg]
