# Supplementary material for: LIPH contributes to glycolytic phenotype in pancreatic ductal adenocarcinoma by activating LPA/LPAR axis and maintaining ALDOA stability
Source: J Transl Med. 2023 Nov 21;21:838. doi: 10.1186/s12967-023-04702-6 (PMC10664664; doi:10.1186/s12967-023-04702-6)
Supplement: Supplementary file 7 — Additional file 7: Table S1. The information of the 10 genes in Veen. [file 12967_2023_4702_MOESM7_ESM.docx]

**Additional file 7: Table S1**

| **TABLE1** \| The information of the 10 genes in Veen | | | | |
| --- | --- | --- | --- | --- |
| Gene Symbol | Function | OS (*P* Value) | HR | DEG along malignant progression (CRA001160) |
| COL17A1 | A structural component of hemidesmosomes, multiprotein complexes at the dermal-epidermal basement membrane zone that mediate adhesion of keratinocytes to the underlying membrane. | 0.00039 | 2.1 | NO |
| SLC2A1 | This gene encodes a major glucose transporter in the mammalian blood-brain barrier. | 0.0043 | 1.8 | NO |
| CDH3 | Calcium-dependent cell-cell adhesion protein. | 0.027 | 1.6 | NO |
| STYK1 | Play important roles in diverse cellular and developmental processes, such as cell proliferation, differentiation, and survival. | 0.026 | 1.6 | NO |
| KCNN4 | Potentially heterotetrameric voltage-independent potassium channel that is activated by intracellular calcium. | 0.000069 | 2.3 | NO |
| RHBDL2 | The encoded protein is thought to release soluble growth factors by proteolytic cleavage of certain membrane-bound substrates, including ephrin B2 and ephrin B3. | 0.021 | 1.6 | NO |
| GPR87 | A G protein-coupled receptor and is located in a cluster of G protein-couple receptor genes on chromosome 3. | 0.0078 | 1.8 | NO |
| LIPH | Catalyzes the production of LPA, which is a lipid mediator with diverse biological properties that include platelet aggregation, smooth muscle contraction, and stimulation of cell proliferation and motility. | 0.004 | 1.8 | YES |
| AHNAK2 | Play a role in calcium signaling by associating with calcium channel proteins. | 0.0063 | 1.8 | NO |
| MST1R | A cell surface receptor for macrophage-stimulating protein (MSP) with tyrosine kinase activity. | 0.029 | 1.6 | NO |

*OS: Overall Survival; HR: Hazard ratio (The clinical prognosis is collected from GEPIA based on the PAAD-TCGA database);

*DEG: Differential gene expression (LogFC $\geq$1); The results of DEG along malignant progression are based on Monocle2 analysis to CRA00160.
