# Supplementary material for: LIPH contributes to glycolytic phenotype in pancreatic ductal adenocarcinoma by activating LPA/LPAR axis and maintaining ALDOA stability
Source: J Transl Med. 2023 Nov 21;21:838. doi: 10.1186/s12967-023-04702-6 (PMC10664664; doi:10.1186/s12967-023-04702-6)
Supplement: Supplementary file 8 — Additional file 8. Extended information. [file 12967_2023_4702_MOESM8_ESM.docx]

| REAGENT | SOURCE | IDENTIFIER | CONCENTRATION |
| --- | --- | --- | --- |
| Antibodies |  |  |  |
| HK2 | Abclonal | Lot: A0994 | WB: 1:1000; IHC: 1:100 |
| GLUT1 | Abclonal | Lot: A6982 | WB: 1:1000; IHC: 1:100 |
| CTGF | Abclonal | Lot: A11456 | WB: 1:1000; IHC: 1:100 |
| UBE2O | Abclonal | Lot: A10036 | WB: 1:1000 |
| P-AKT | CST | Lot: 4060T | WB: 1:1000; IHC: 1:50 |
| AKT | CST | Lot: 9272S | WB: 1:1000 |
| p-ERK | CST | Lot: 4370T | WB: 1:1000 |
| ERK | CST | Lot: 4695T | WB: 1:1000 |
| GAPDH | Proteintech | Lot: 60004-1-lg | WB: 1:2000 |
| ACTB | Proteintech | Lot: 20536-1-AP | WB: 1:2000 |
| YAP | CST | Lot: 14074S | WB: 1:1000; IF: 1:50; ChIP: 1:50 |
| p-YAP (ser127) | CST | Lot: 13008T | WB: 1:1000 |
| Bax | CST | Lot: 41162S | WB: 1:1000 |
| HIF-1α | CST | Lot: 36169T | WB: 1:1000 |
| LIPH | Proteintech | Lot: 16602-1-AP | IF: 1:50 |
| LIPH | abcam | Lot: ab192615 | WB: 1:1000; IHC-P: 1:100 |
| Flag | CST | Lot: 14793S | WB: 1:1000; IP: 1:50 |
| ALDOA | Proteintech | Lot: 11217-1-AP | WB: 1:1000; IHC: 1:100; IF: 1:1000 |
| Lamin B1 | Proteintech | Lot: 12987-1-AP | WB: 1:1000 |
| Ki67 | Servicebio | Lot: GB121499 | IHC: 1:500 |
| Goat-anti-rabit | Proteintech | Lot: 20000311 | WB: 1:5000 |
| Anti-Mouse-IgG | Proteintech | Lot: 20000261 | WB: 1:5000 |
| Goat Anti-Mouse IgG H&L (Alexa Fluor® 488) | abcam | Lot: ab150113 | IF: 1:100 |
| IgG (H+L) (Cy3 conjugated Goat Anti-Rabbit IgG (H+L)) | Servicebio | Lot: GB21303 | IF: 1:100 |
| IgG (H+L) (Cy5 conjugated Goat Anti-mouse IgG (H+L)) | Servicebio | Lot: GB27301 | IF: 1:100 |

| REAGENT | SOURCE | DETAILs |
| --- | --- | --- |
| Plasmids |  |  |
| sh1-LIPH | Bioegene | GGGUUUGCUCUCUGUUGAAGATT  UCUUCAACAGAGAGCAAACCCTT |
| sh3-LIPH | Bioegene | GGAACUUGAAUGUGACCAAGATT  UCUUGGUCACAUUCAAGUUCCTT |
| Primers |  |  |
| GLUT1-F  GLUT1-R | Sangon | ATTGGCTCCGGTATCGTCAA  GCTCAGATAGGACATCCAGGGTA |
| HK2-F  HK2-R | Sangon | AGCCCTTTCTCCATCTCCTT  GCTTGCCTACTTCTTCACGG |
| ENO1-F  ENO1-R | Sangon | GCCGTGAACGAGAAGTCCTG  ACGCCTGAAGAGACTCGGT |
| TPI1-F  TPI1-R | Sangon | AGCTCATCGGCACTCTGAAC  CCACAGCAATCTTGGGATCT |
| PDK1-F  PDK1-R | Sangon | CTGTGATACGGATCAGAAACCG  TCC ACCAAACAATAAAGAGTGCT |
| LIPH-F  LIPH-R | Sangon | GGAACTTGAATGTGACCAAGA  TTTCCTTGTGTAGAGCATCAGC |
| CTGF-F  CTGF-R | Sangon | ACCGACTGGAAGACACGTTTG  CCAGGTCAGCTTCGCAAGG |
| AREG-F  AREG-R | Sangon | CCCCAAAACAAGACGGAAAGTG  CCCACACCGTTCACCGAAATA |
| ID1-F  ID1-R | Sangon | CTGCTCTACGACATGAACGG  GAAGGTCCCTGATGTAGTCGAT |
| YAP1-F  YAP1-R | Sangon | CCCTCGTTTTGCCATGAACC  GTTGCTGCTGGTTGGAGTTG |
| TAZ-F  TAZ-R | Sangon | CACCGTGTCCAATCACCAGTC  TCCAACGCATCAACTTCAGGT |
| DIAPH1-F  DIAPH1-R | Sangon | CGACGGCGGCAAATCTAAGAA  CAGGTTCATATCCAGCAGCATC |
| ACTB-F  ACTB-R | Sangon | GAGCACAGAGCCTCGCCTTT  TCATCATCCATGGTGAGCTGG |
| GAPDH-F  GAPDH-R | Sangon | ACAGTCAGCCGCATCTTCTTT  CAATACGACCAAATCCGTTGACT |
| HIF1AP-F  HIF1AP-R | Tsingke | TCGTTGCTCAGATGTGTTAC  CTTAGTAGACAAGGTGAGTTCC |
